# A new framework with convoluted oscillatory neural network for efficient object-based land use and land cover classification on remote sensing images

**DOI:** 10.3389/frai.2025.1696859

**Published:** 2025-12-17

**Authors:** Chirag Jitendra Chandnani, Shlok Chetan Kulkarni, Geraldine Bessie Amali D, Rohini Selvaraj

**Affiliations:** School of Computer Science and Engineering, Vellore Institute of Technology, Vellore, Tamil Nadu

**Keywords:** convolution neural network, LANDSAT-8, remote sensing, oscillatory functions, land use land cover (LULC)

## Abstract

Rigorous urbanization leads to unprecedented climate change. Pune area in India has witnessed recent flash floods and landslides due to unplanned rapid urbanization. It, therefore, becomes vital to manage and analyse man-made impact on the environment through effective land use land cover classification (LULC). Accurate LULC classification allows for better planning and effective allocation of resources in urban development. Remote sensing images provide surface reflectance data that are used for accurate mapping and monitoring of land cover. Convolution neural networks (CNN) trained with Relu are conventionally used in classifying different land types. However, every neuron has a single hyperplane decision boundary which restricts the model's capability to generalize. Oscillatory activation functions with their periodic nature have demonstrated that a single neuron can have multiple hyperplanes in the decision boundary which helps in better generalization and accuracy. This study proposes a novel framework with convoluted oscillatory neural networks (CONN) that synergistically combines the periodic, non-monotonic nature of oscillatory activation functions with the deep convoluted architecture of CNNs to accurately map LULC. Results carried out on LANDSAT-8 surface reflectance images for the Pune area indicate that CONN with Decaying Sine Unit achieved an overall train accuracy of 99.999%, test accuracy of 95.979% and outperforms conventional CNN models in precision, recall and User's Accuracy. A thorough ablation study was conducted with various subsets of the feature set to test the performance of the selected models in the absence of data.

## 1 Introduction

Remote sensing technologies provide valuable information for observing and analyzing the Earth's surface. More importantly, these data monitor environmental changes, including climate change, urbanization, and natural disasters, such as landslides (Kamusoko, 2017). Without remote sensing data, monitoring these environmental changes would be very difficult, and it requires extensive human resources. Land use land cover (LULC) classification provides detailed information regarding land cover and land usage patterns in specific regions (Attri et al., 2015). Accurate LULC classification is essential for urban planning, supporting urban development with environmental sustainability, thereby mitigating conflicts between the two. With rapid urbanization, cities such as Pune require accurate LULC data to address and minimize land degradation. Remote sensing involves gathering data through different approaches, including the use of satellites, aerial vehicles, and aircraft-mounted cameras. It helps to provide the necessary information for LULC classification in the Pune area (Fu et al., 2020). Among them, LANDSAT, MODIS, and Sentinel satellites are Earth observation satellites, which provide remote sensing data for periodic monitoring of LULC changes (Zhao et al., 2022). Sentinel-2 has a fast revisit time and high spatial resolution. Very high resolution (VHR) sensors like WorldView-3 and the Gaofen (GF) series provide complex urban detail, often at 0.8–1.2 m resolution (Pal et al., 2023) However, these VHR datasets pose major problems as they are commercial and expensive, which complicates large-area analysis (Wei et al., 2023). Furthermore, Sentinel-2 has been proven to be less accurate than Landsat-8 for certain LULC classifications (Kumari and Karthikeyan, 2023). Landsat's superiority over Sentinel-2 lies in its cost efficiency, and continuous long-term historical analysis. Researchers frequently rely on LANDSAT datasets for accurate observation and analysis, given their high spatial and temporal resolution. With its 30-m spatial resolution, it supports detailed monitoring of surface features and their variations over time. In addition, it supports the identification of subtle features, including urban development, agricultural patterns, and shifts in forest ecosystems (Hansen and Loveland, 2012). Substantial computational resources and specialized software are needed to process this high-resolution data. Google Earth Engine (GEE) provides a cloud-based platform with access to extensive datasets and computational power (Amani et al., 2020). The LULC classification process within GEE involves data pre-processing, feature extraction, and classification using machine learning algorithms. This process can be carried out using two main methods: pixel-based and object-based approaches. In the pixel-based method, individual pixels are treated as standalone entities, with features extracted solely from each pixel without considering any surrounding context. In contrast, the object-based method converts pixels into larger objects, allowing for feature extraction from these objects. This strategy utilizes spatial, spectral, and contextual inputs to achieve a comprehensive analysis of LULC patterns (Dingle Robertson and King, 2011). Some of the main object-based methods include simple linear iterative clustering (SLIC) and simple non-iterative clustering (SNIC) (Achanta et al., 2012; Achanta and S'´usstrunk, 2017). Using SNIC for image segmentation improves classification accuracy by grouping pixels with similar characteristics into clear segments. SNIC's non-iterative method can be faster because it skips the repeated steps found in SLIC, which may result in quicker processing times. This approach helps in creating more defined and accurate image classifications (Selvaraj and Amali, 2024). To effectively classify these extracted features, a variety of machine learning algorithms, including random forest, support vector machines, and Naive Bayes, are employed. However, issues including algorithm selection, parameter adjustment, and susceptibility to overfitting can influence the precision and robustness of the final LULC classification (Talukdar et al., 2020).

Several studies (Nigar et al., 2024) have highlighted the benefits of Deep Learning (DL) over traditional ML for LULC classification due to DL's automatic feature extraction and ability to handle complex and generalized, high-dimensional data. Among DL techniques, CNNs have proven highly effective, as they can extract features without manual intervention and enhance classification precision across multiple image datasets (Zhao et al., 2023; Teja et al., 2024).

**Figure 1:**
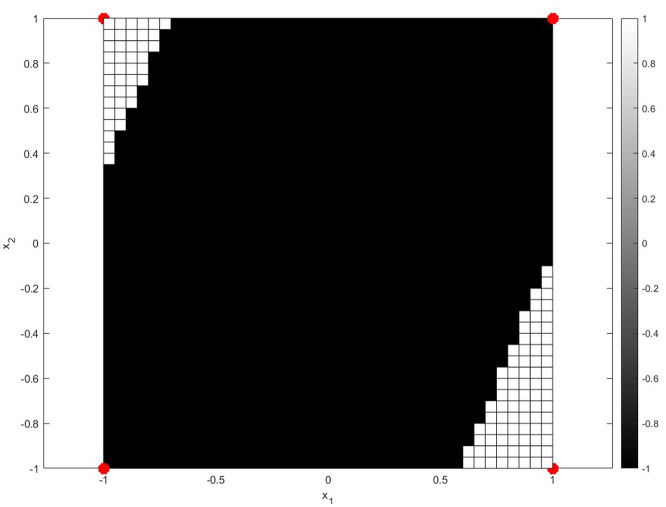
The XOR problem learnt by a single neuron using the DSU activation function.

The strength of DL lies in its ability to capture intricate feature representations, which has led to superior results across diverse classification applications such as LULC classification. Among DL models, CNNs in particular have emerged as the most effective for imagebased classification tasks. CNNs excel in image processing and analysis as they leverage their convolutional layers to detect spatial features which help the model learn patterns efficiently. In contrast to standard machine learning algorithms that rely on manual feature collection, CNNs automatically learn patterns such as edges, textures, and object shapes thus making them suitable for RS based use cases. However, despite their advantages, DL models face certain challenges. The vanishing gradient problem represents a significant drawback, as shrinking gradients during backpropagation can result in stalled or very slow learning in deep architectures. This issue is particularly pronounced in deep CNNs using traditional activation functions like Sigmoid and Tanh, which reduce the inputs to a narrow range thereby limiting the gradient flow. To address this, recent research has proposed alternative oscillatory activation functions that enhance gradient propagation and improve model performance. Oscillating activation functions offer a distinct advantage in deep learning tasks that involve complex, repetitive patterns by leveraging their periodic nature (Noel et al., 2025b). Unlike traditional functions like ReLU and sigmoid, which can suffer from saturation (where gradients vanish and learning stagnates), oscillating functions maintain consistent gradient flow, enabling faster convergence and better performance on nonlinear separable (Noel et al., 2025b). Bio-inspired oscillating activation functions functionally bridge the performance gap between biological and artificial neurons. With the recent discovery of special human neocortical pyramidal neurons that can individually learn the XOR function the fundamental performance gap is clear, as it is impossible for single artificial neurons using traditional activation functions to learn the XOR function (Noel et al., 2025a). The XOR function is significant because it requires the neuron to learn a non-linearly separable data. Traditionally, two hyperplanes are required for class separation, meaning activation functions must have a minimum of two zeros to solve this problem with a single neuron. However, popular activation functions like sigmoid, ReLU, Swish, and Mish are single-zero activation functions which means they have a single zero at origin and thus cannot solve the XOR function individually (Noel et al., 2025a). They are limited to only linear classification. The biological neurons in layers two and three of the human cortex have oscillating activation functions which first increase to a maximum with input and then decrease. Thus, being able to learn the XOR function individually. Biologically inspired Oscillating activations due to their multiple zero boundaries allow a single artificial neuron to divide its input space with multiple hyperplanes like a biological neuron, enabling it to make more complex, non-linear decisions (Noel et al., 2025a). A single neuron enhanced with oscillating activation functions can learn the XOR problem. Oscillating activation functions such as shifted sinc unit (SSU) and decaying sine unit (DSU) have a derivative of 1 at the origin. This helps learning quickly when weights are initialized to small values. Due to their higher representative power, classification problems can be solved with fewer neurons and fewer layers than required by networks using popular activation functions. Furthermore, they are non-saturating for all inputs thus they do not suffer from the vanishing gradient problem prevalent in the traditional activation functions. The XOR problem involves learning the following dataset (Figure 1):


D={([-1,-1],-1),([1,-1],1),([-1,1],1),([1,1],-1)}

The XOR problem was solved using a single neuron with oscillatory activation functions, mean-square loss, and simple Stochastic Gradient Descent (SGD). A learning rate of α = 0.1 and the SGD update rule


$$\Delta \text{w}=\alpha \left(y-g\left(z\right)\right)g^{\prime }\left(z\right)\text{x},\;z={\text{w}}^{T}\text{x}+b$$

was used. The initial weight vector **w** was initialized with uniform random numbers in the interval [−1, 1]. The XOR function was successfully learned by a single neuron with activation function *g*(*z*) chosen as:


$$g\left(z\right)=\frac{\pi }{2}\left(\text{sinc}\left(z-\pi \right)-\text{sinc}\left(z+\pi \right)\right)$$

The target *y* for each input was taken as the class label, namely 1 or −1. After training, the output of the neuron is mapped to the class label in the usual manner. Positive outputs are mapped to +1 and negative outputs to −1. This is done by using a signum function defined as:


$$\text{sign}(z)=\{\begin{array}{ll} 1 & \text{if }z>0\\ -1 & \text{if }z<0\\ 0 & \text{if }z=0 \end{array}$$

The study explores the integration of oscillatory activation functions in deep convolutional architectures, along with SNIC and GLCM for LULC classification. Unlike traditional activation functions, oscillatory activation functions, are known to maintain a more consistent gradient flow attributed to their periodic nature, potentially addressing critical training limitations such as the vanishing gradient problem and slow convergence speed. The work proposes Convoluted Oscillatory Neural Networks (CONN), in which we aim to optimize feature extraction and model accuracy. Through the integration of SNIC and GLCM textural features, the framework is optimized to enhance both feature representation and classification accuracy. The main focus is to propose an efficient and reliable LULC classification technique for the city of Pune using publicly available remote sensing data, specifically LANDSAT-8 images, classifying the region into four classes: Water, Urbanized, Vegetation and Barren land (Xie et al., 2021; Digra et al., 2022) and emphasizing the Surface Reflectance imagery (Whiteside and Ahmad, 2005).

**Figure 2:**
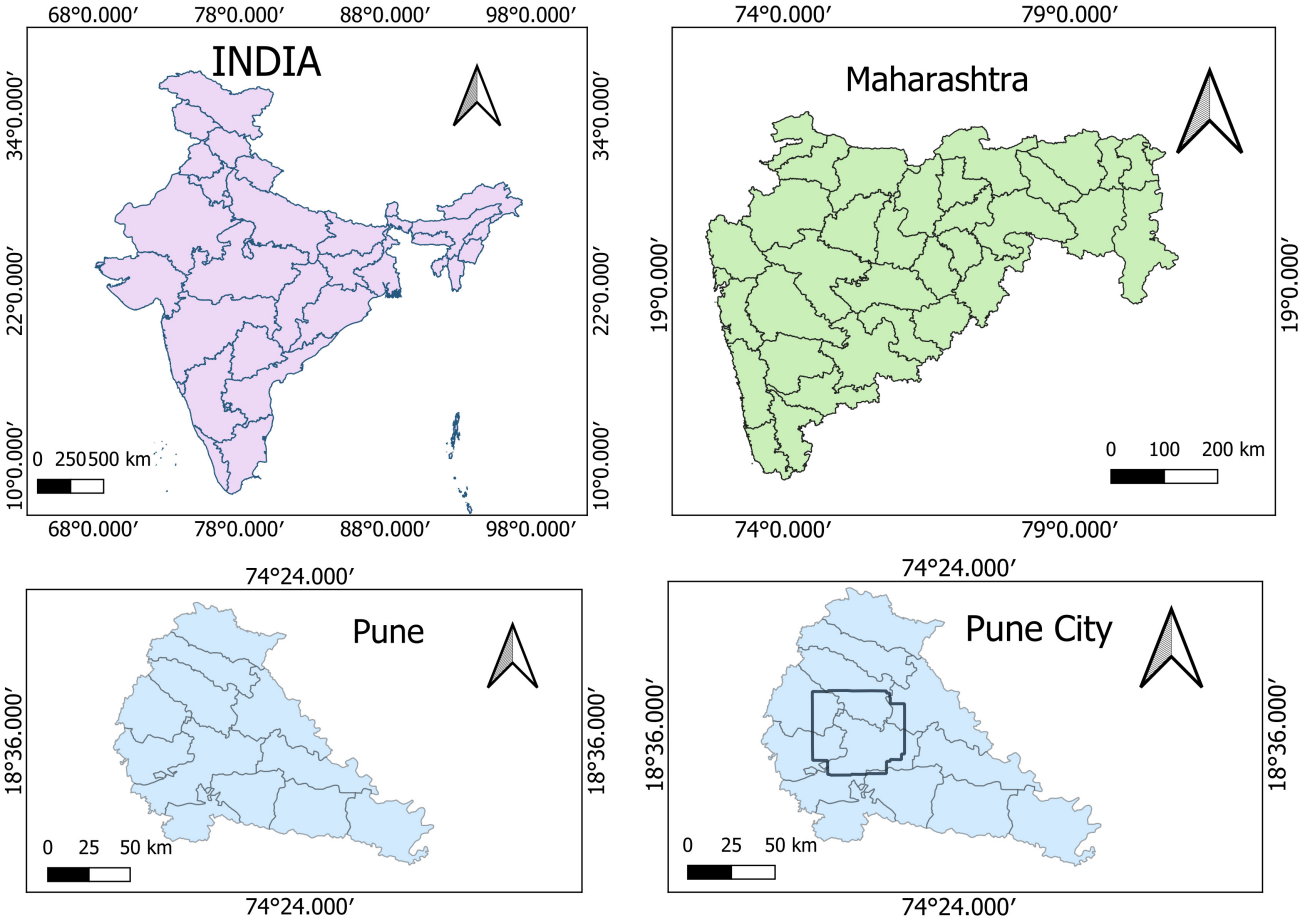
Study area—Pune.

**Figure 3:**
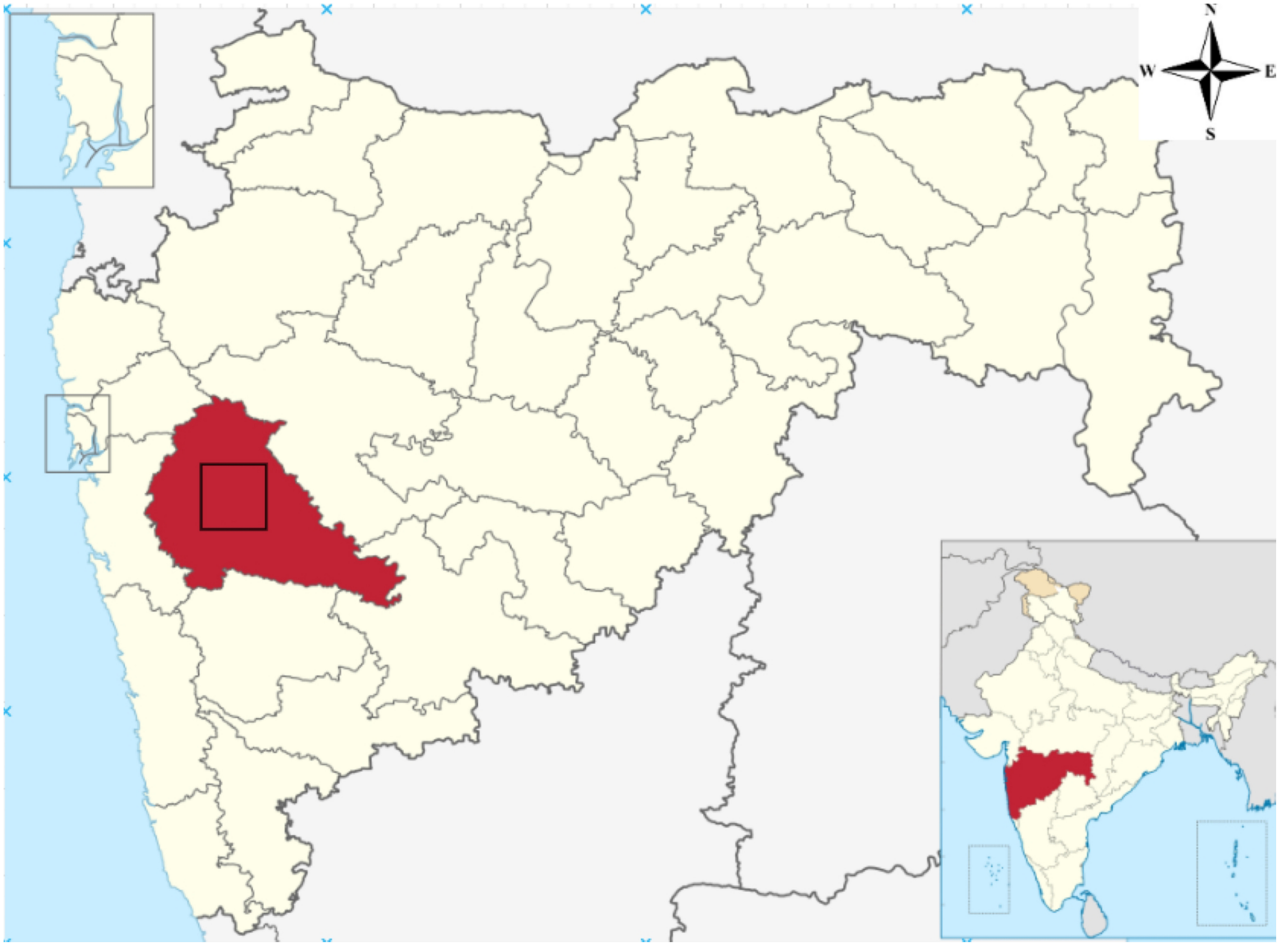
Pune region geometry.

The Pune Metropolitan Region in western India covers around 5,052 km^2^, with a population of over 7.18 million (see Figures 2, 3). It presents a wide variety of features, such as forests, lakes, and farmland. Additionally, in recent years, it has showcased a high rate of urbanization (Census of India, 2024; Maharashtra Government, 2020). This high rate of urbanization is alarming as it poses a threat of environmental degradation, resource exhaustion, and even the breakdown of the natural ecosystems (Padigala, 2012). This rapid transition has already been shown to have a negative impact on the temperature, rainfall patterns, and even hydrological cycles in the region (Sandbhor et al., 2022). However, a lack of accurate and current land cover data makes it harder to manage and protect the region effectively (Samuel and Atobatele, 2019). The region, therefore, serves as an excellent candidate for the purposes of this study.

The remainder of the study is organized as follows: Section 2 contains the proposed methodology which includes a detailed explanation of the proposed methodology, including the dataset, pre-processing techniques, and the models used. Results and Ablation Study section discusses the findings, compares them with previous works, and highlights key insights inferred from the model output. Finally, Conclusion and Future Works concludes the study by summarizing the contributions and outlining potential future research directions.

## 2 Materials and methods

**Figure 4:**
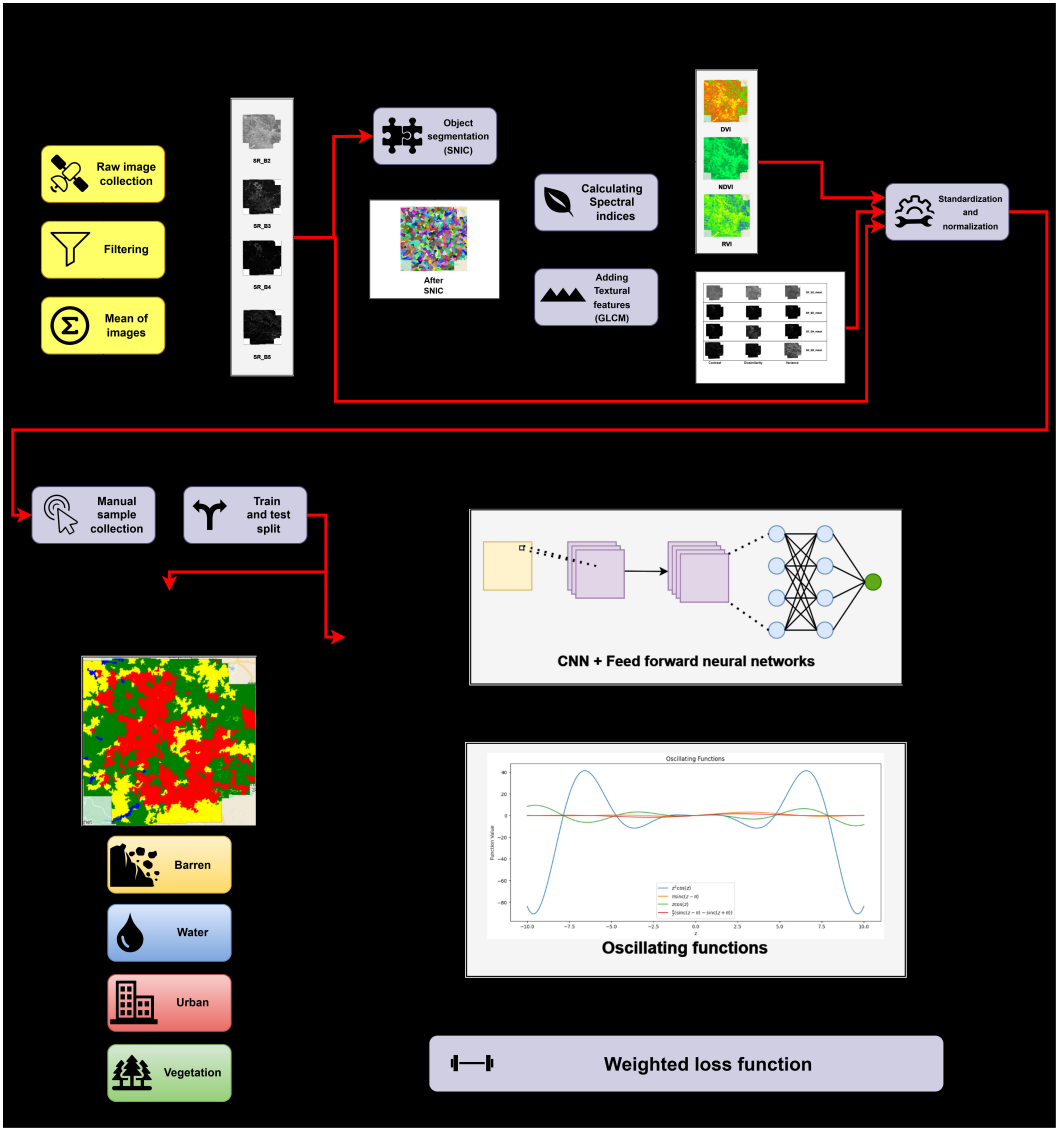
Workflow diagram for the proposed methodology.

This section, as shown in Figure 4, discusses the steps—data collection, SNIC segmentation, feature selection and engineering, data pre-processing, model training, and finally, the metrics and hardware and software setup utilized.

### 2.1 Data collection

This study utilizes the “Landsat 8 Level 2 Tier 2 Collection 2” SR images that are already geometrically and atmospherically corrected (Markham and Barsi, 2017). Another major advantage of the dataset is the near-continuous availability of data. With Landsat-8 and 9 updating data once every 16 days, it is possible to have regular monitoring of land use patterns. Furthermore, while Landsat-9 data is of higher radiometric resolution, this study utilizes a Landsat-8 data collection to strike a balance between computational load and LULC classification accuracy.

**Figure 5:**
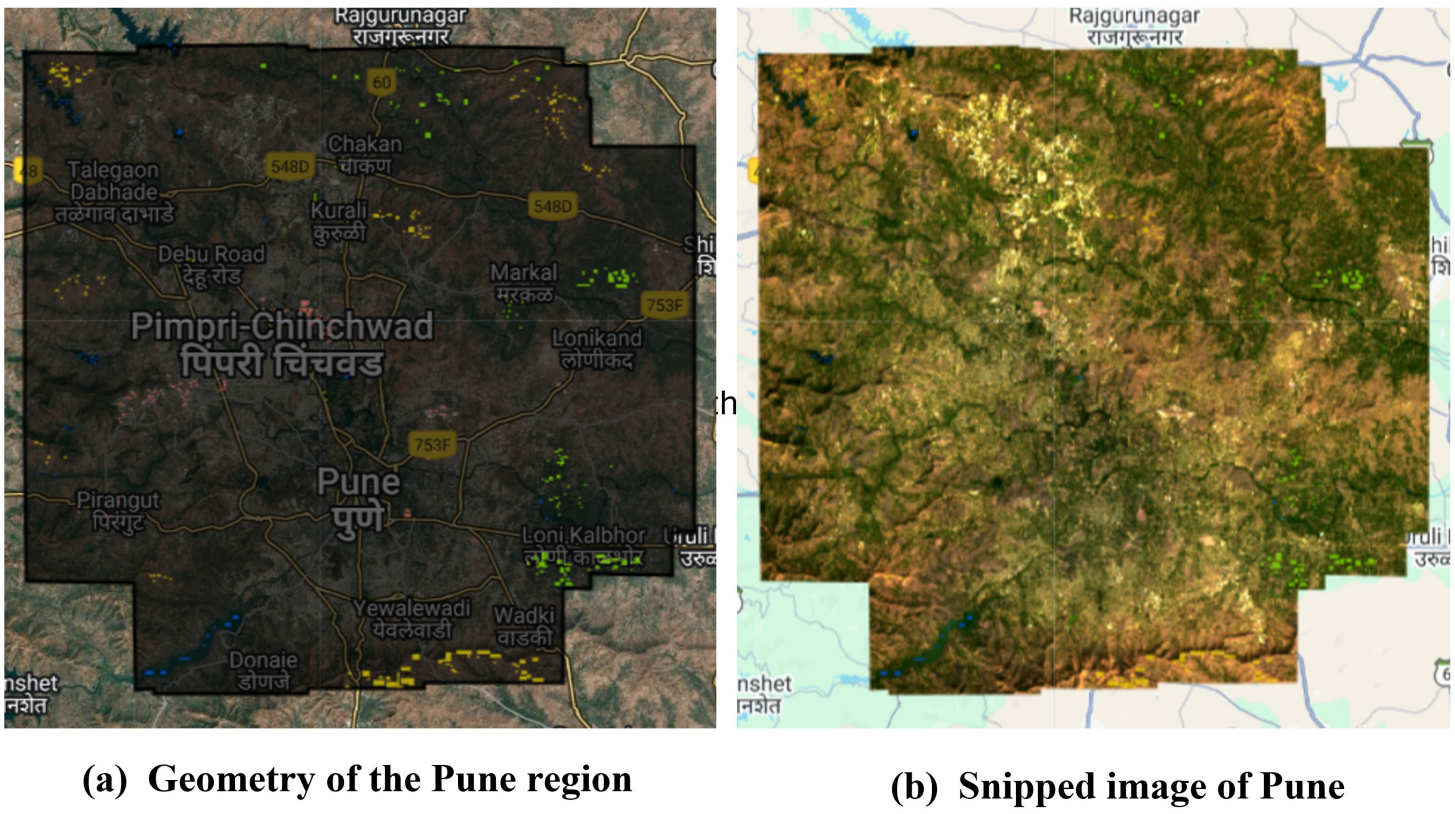
Process of extracting the image for Pune region from Landsat 8 imagery. **(a)** Geometry of the Pune region and **(b)** snipped image of Pune.

The images in the aforementioned image collection consist of four visible bands, 1 Near-Infrared band (NIR), and two Short-Wave Infrared bands (SWIR). Additionally, the quality assessment (QA) band in the SR collection makes it easier to filter out cloud cover. From the aforementioned bands available in the image collection, this study utilizes bands *SRB*_5, *SRB*_4, *SRB*_3, and *SRB*_2. Each of these bands provides essential information that helps in distinguishing various land cover types, including urban areas, vegetation, and water bodies (U.S. Geological Survey, 2019). Their specific uses are provided in Table 1. Finally, a masked image of the study area for the selected features was obtained using the geometry as shown in Figure 5. Furthermore, a mean composite was calculated for all the images in the region over the selected time period. This was chosen to be from January 2024 to February 2024, as this time period was observed to have the lowest cloud cover, eliminating the need for complex image corrections. Table 2 summarizes the important input parameters required for data insertion in GEE. This image is then used to create objects using SNIC segmentation.

**Table 1:** Significance of selected spectral features with wavelength information.

**Spectral feature**	**Significance**	**Wavelength**
*SRB*_5 (NIR)	Sensitive to biomass; useful for identifying vegetation	0.851–0.879 μm
*SRB*_4 (Red)	Helps distinguish vegetation from non-vegetation; used in vegetation indices	0.636–0.673 μm
*SRB*_3 (Green)	Identifies water and vegetation	0.533–0.590 μm
*SRB*_2 (Blue)	Distinguishes urban areas from water bodies	0.452–0.512 μm

**Table 2:** Important input parameters for data insertion in GEE.

**Important input parameters and code for data insertion in GEE**	
• Region of interest (geometry boundary of the study area) • The time period of interest (starting and ending time frame) • Cloud coverage filter (less than 10% of cloud coverage)	Collection = “LANDSAT/LC08/C02/T1_L2”; Filter date: 2024-01-01 to 2024-02-28; Filter geometry: geometry; Filter cloud cover: less than 10%

### 2.2 Object-based segmentation using simple non-iterative clustering (SNIC)

Object-based segmentation forms an integral part of this study. The inclusion of object-based segmentation ensures that the model is less susceptible to noise and avoids other issues associated with pixel-based segmentation techniques, like the “salt and pepper effect” (Ouchra et al., 2022). For the task of object-based segmentation, this study utilizes SNIC as it has shown to be effective in previous literature (Whiteside and Ahmad, 2005). Algorithm 1 depicts the SNIC algorithm. Firstly, the label map *L* is created with all zeros. Following that, the priority queue and seed elements are initialized. SNIC is initialized with *k* initial centroids which are spaced *size* pixels apart, where *k* is a user-defined parameter and *size* is calculated using *k* and the dimensions of the image. All these seed elements are then pushed into the priority queue *Q*, with each element containing values of position, color, label, and distance. In the second stage i.e. the propagation stage, the element in the priority queue with the smallest distance, *e*_*i*_, is popped. This distance is calculated using Equation 1. If the element is not yet assigned to a centroid in the label map, it is then assigned to centroid *k*_*i*_. After this update is done, the centroid *k*_*i*_ itself is updated to incorporate the new element using online averaging. The algorithm then proceeds to check all connected neighbors of *e*_*i*_ and assign them the label *k*_*i*_. If not already labeled on the label map, these elements are pushed onto *Q*. This is repeated until *Q* is empty, and the label map *L* will be returned as a result.


$$\begin{array}{l} d=\sqrt{{\left(c_{j}-c_{k}\right)}^{2}+{\left(\frac{m}{s}\right)}^{2}{\left(x_{j}-x_{k}\right)}^{2}} \end{array}$$ (1)

where:

- *c*_*j*_,*c*_*k*_: Color vectors in CIELAB/Lab* space
- *x*_*i*_,*x*_*j*_: Spatial coordinates
- *m*: compactness parameter
- *s*: initial grid spacing

Algorithm 1 SNIC Segmentation Algorithm

1:  Input: Input image *I*, *K* initial centroids *C*[*k*] = {*x*_*k*_, *c*_*k*_} sampled on a regular grid, color normalization factor *m*
2:  Output: Assigned label map *L*
3:  Initialize *L*[:]←0
4:  for *k*←1 **to** *K* **do**
5:   Element *e*←{*x*_*k*_, *c*_*k*_, *k*, 0}
6:   Push *e* on priority queue *Q*
7:  end **for**
8:  while *Q* is not empty **do**
9:   Pop *Q* to get *e*_*i*_
10:   if *L*[*x*_*i*_] = 0 **then**
11:    *L*[*x*_*i*_]←*k*_*i*_
12:    Update centroid *C*[*k*_*i*_] online with *x*_*i*_ and *c*_*i*_
13:    for Each connected neighbor *x*_*j*_ of *x*_*i*_ **do**
14:     Create element *e*_*j*_←{*x*_*j*_, *c*_*j*_, *k*_*i*_, *d*_*j*,_*k*__*i*__}
15:     if *L*[*x*_*j*_] = 0 **then**
16:      Push *e*_*j*_ on *Q*
17:     end **if**
18:    end **for**
19:   end **if**
20:  end **while**
21:  Return *L*

**Figure 6:**
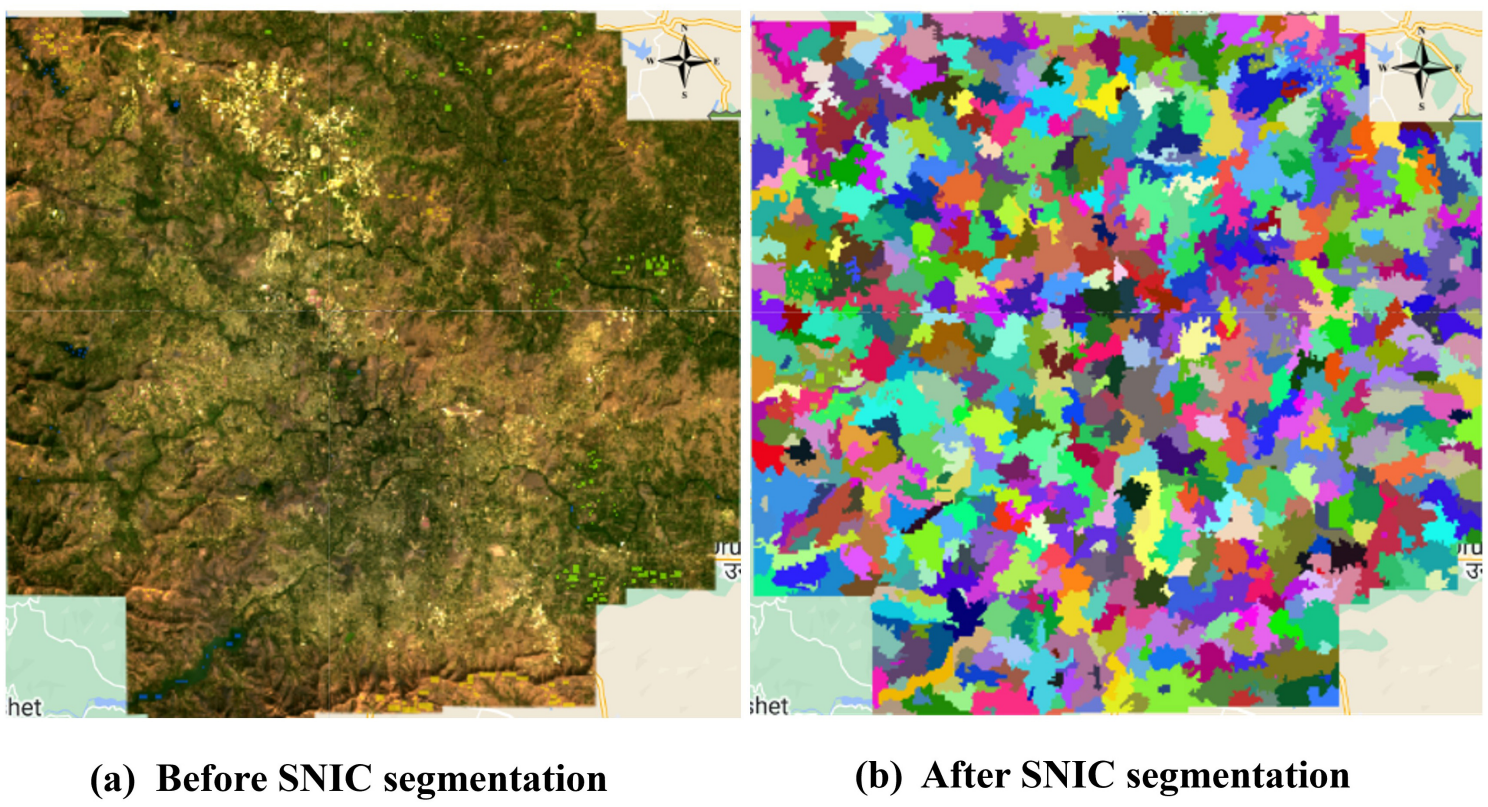
Comparison before and after applying SNIC segmentation. **(a)** Before SNIC segmentation and **(b)** after SNIC segmentation.

The parameters used for the SNIC algorithm and the codes are presented in Table 3. Similarly, the output of the SNIC segmented image can be observed in Figure 6. Post object-based segmentation on the image, feature engineering was performed to calculate additional features.

**Table 3:** SNIC segmentation parameters and code example.

**Parameters**	**Code**
• Image: input image • Size: super pixel seed spacing size • Compactness: shape of the cluster • Connectivity: type of contiguousness to join the cluster	var snicA = ee.Algorithms.Image .Segmentation.SNIC({ image: img, size: 5, compactness: 0.1, connectivity: 4 })

### 2.3 Feature selection and engineering

**Figure 7:**
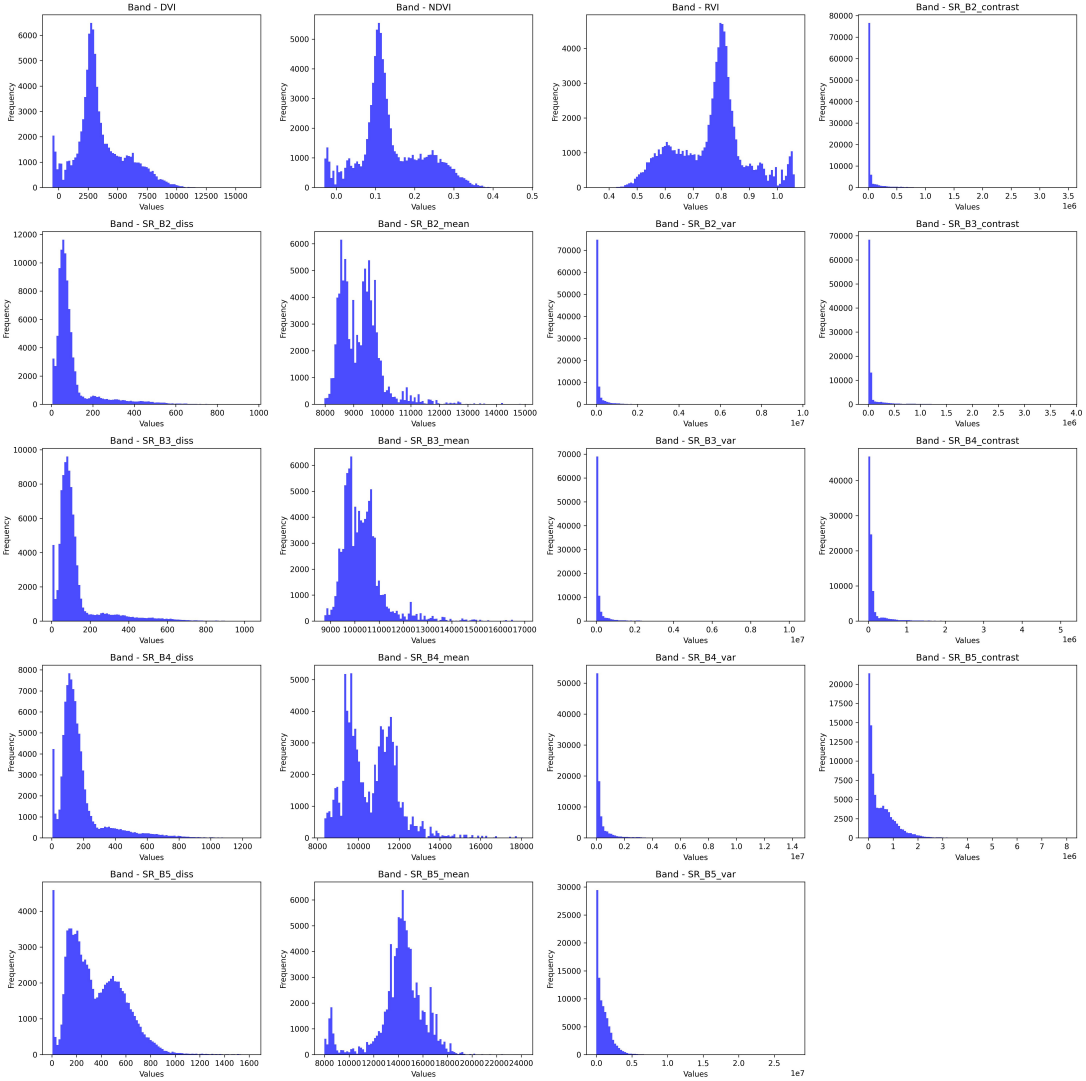
Band frequency visualization for selected bands.

This study utilizes several features extracted from the aforementioned Landsat-8 image collection, each chosen for its relevance to the land cover classification task. This study makes use of textural features obtained using the Gray-Level Co-occurrence Matrix (GLCM) as they have been shown to enhance the classification accuracy for LULC tasks (Haralick et al., 1973). Additionally, this study also utilizes spectral indices like the Normalized Difference Vegetation Index (NDVI), Ratio Vegetation Index (RVI), and Difference Vegetation Index (DVI). The bands selected for this study, along with their descriptions, are provided in Table 4. Furthermore, the frequencies of these bands have been visualized in Figure 7.

**Table 4:** Bands and their applications.

**Index/band**	**Function**	**Mean**	**Standard-deviation**
DVI	Vegetation density (Used for estimating vegetation amount)	3,531.541	2,155.849
NDVI	Vegetation health (Commonly used for monitoring vegetation vigor)	0.138	0.082
RVI	Biomass estimation (Helps in assessing vegetation biomass)	0.766	0.125
SR_B2_contrast	Texture analysis (Band 2—Blue) (used for edge detection and texture classification)	68,104.628	190,161.503
SR_B2_diss	Texture analysis (Band 2—Blue) (useful for land cover differentiation)	100.291	108.227
SR_B2_mean	Blue band reflectance (helps in water body identification and atmospheric correction)	9,233.005	718.388
SR_B2_var	Variability in blue band (useful in studying atmospheric effects)	144,184.107	394,943.885
SR_B3_contrast	Texture analysis (Band 3—Green) (used for detecting land cover texture)	99,397.192	267,888.283
SR_B3_diss	Texture analysis (Band 3—Green) (useful for urban-rural differentiation)	123.994	126.975
SR_B3_mean	Green band reflectance (used for vegetation monitoring and chlorophyll content estimation)	10,294.904	854.736
SR_B3_var	Variability in Green Band (useful for vegetation health assessment)	213,565.510	572,480.607
SR_B4_contrast	Texture analysis (Band 4—Red) (useful in analyzing land cover variation)	163,092.980	367,304.027
SR_B4_diss	Texture analysis (Band 4—Red) (used for land classification and change detection)	175.269	149.412
SR_B4_mean	Red band reflectance (used for vegetation stress and soil background reflectance)	10,626.561	1,269.153
SR_B4_var	Variability in red band (helpful for crop health monitoring)	366,543.304	803,721.235
SR_B5_contrast	Texture analysis (Band 5—NIR) (useful for biomass structure analysis)	523,451.902	644,512.645
SR_B5_diss	Texture analysis (Band 5—NIR) (used for vegetation cover differentiation)	356.837	224.516
SR_B5_mean	Near-infrared reflectance (vegetation health) (key for identifying healthy vegetation and water stress)	14,183.855	2,033.679
SR_B5_var	Variability in near-infrared band (useful for detecting forest degradation and biomass estimation)	1,097,093.532	1,527,726.299

#### 2.3.1 Vegetation indices (spectral indices)

Vegetation indices refer to mathematical transformations of the spectral bands. They are used to better visualize and identify vegetation and can, therefore, be used to boost the efficacy of LULC tasks. This study utilizes NDVI, RVI, and DVI. Each of these indices uses different spectral bands for their calculation, offering distinct perspectives on vegetation properties and enabling targeted insights into land cover characteristics (as shown in Table 5).

**Table 5:** Descriptions of vegetation indices used in this study.

**Spectral Index**	**Formula**	**Description**	**Image**
DVI	DVI = NIR − Red	DVI is a simpler version of NDVI and provides an absolute measure of vegetation presence	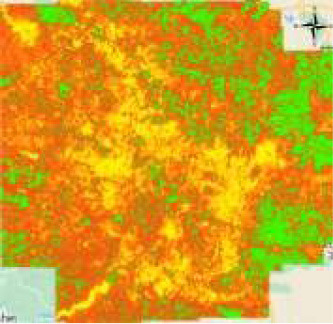
NDVI	$\text{NDVI}=\frac{\text{NIR}-\text{Red}}{\text{NIR}+\text{Red}}$	NDVI measures vegetation health and density. High NDVI values indicate dense, healthy vegetation, while low values indicate barren land, water, or built-up areas	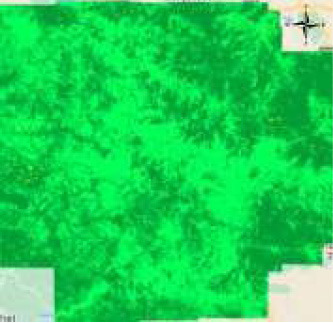
RVI	$\text{RVI}=\frac{\text{NIR}}{\text{Red}}$	Ratio between NIR and Red reflectance rather than the difference. It is more sensitive to areas with low vegetation cover.	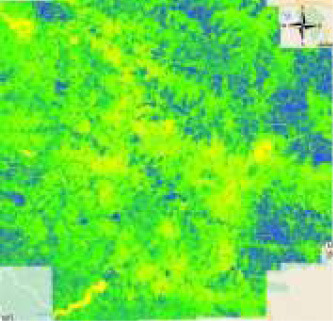

#### 2.3.2 Textural features

In addition to spectral features and spectral indices, this study also utilizes GLCM to generate textural features for the study area. GLCM produces a total of 18 features for each band present in the input image, however, due to computational constraints, this study has selected the following three features that were found to be the most relevant to the task at hand.

##### 2.3.2.1 Contrast

**Figure 8:**
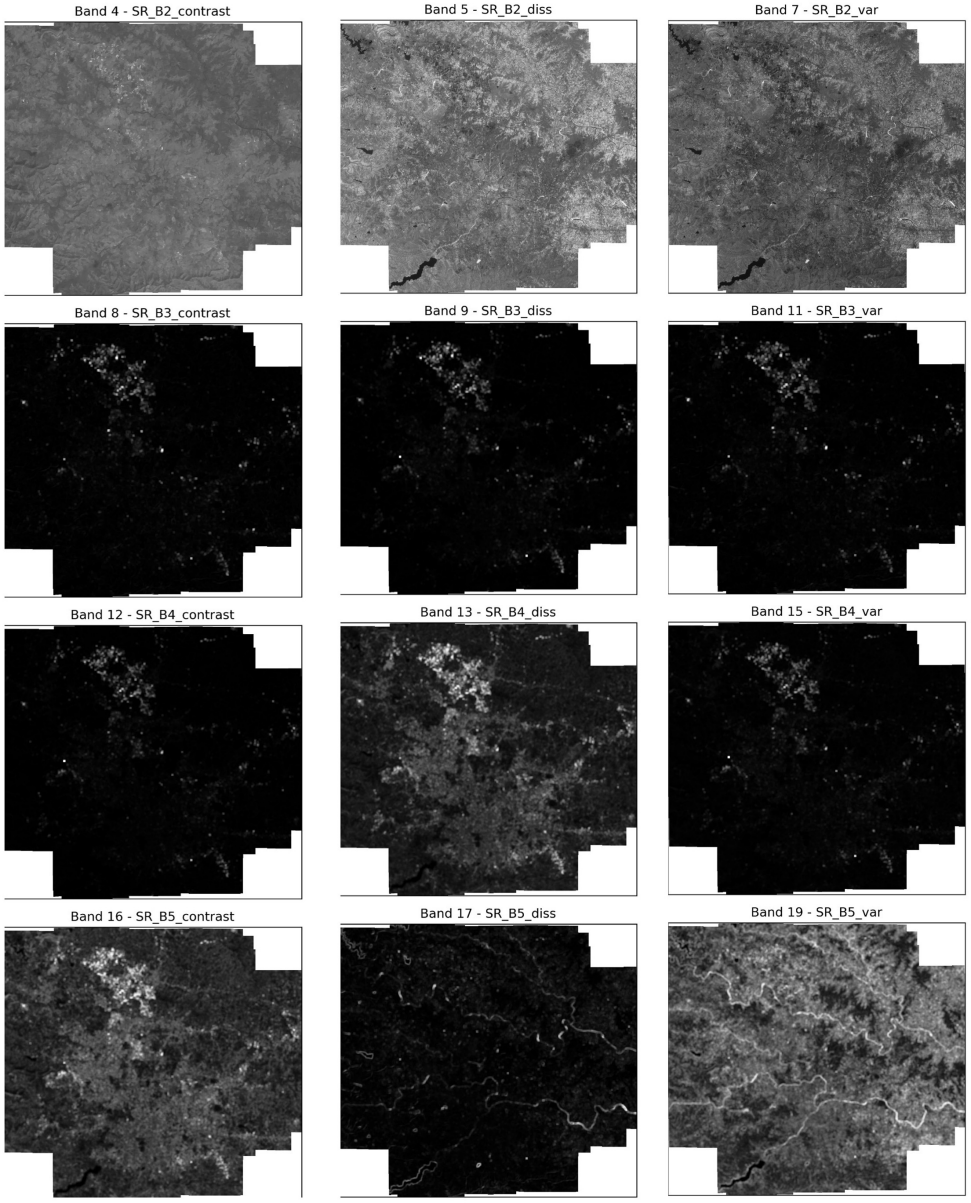
GLCM contrast, dissimilarity, variance bands.

The contrast feature of GLCM captures the difference between the intensities of neighboring pixels. This feature is especially useful for detecting similar materials with different surface textures. This ability is paramount for the LULC task, as high contrast values usually indicate important landscape features such as water, vegetation, urban, etc. The depiction of GLCM contrast features for each of the selected bands can be observed in Figure 8.

##### 2.3.2.2 Variance

The variance feature in GLCM represents the difference between the intensities of pixels in a spatial neighborhood. A high variance indicates a change in land cover, such as vegetation health or soil moisture. Moreover, variance is more resistant to noise in the data and hence is more suited for diverse regions. The depiction of GLCM variance features for each of the selected bands can be observed in Figure 8.

##### 2.3.2.3 Dissimilarity

The dissimilarity feature in GLCM represents the dissimilarity or difference of a pixel as compared to its neighboring pixels. This feature lends itself to the detection of edges, which is essential in LULC classification. The depiction of GLCM dissimilarity features for each of the selected bands can be observed in Figure 8.

### 2.4 Data preprocessing–Using normalization

Pre-processing is a fundamental step for training machine and deep learning models. It boosts data quality by tending to issues like noise, lost values, and irregularities. This is essential for superior model performance and precision. Alongside irregular data patterns, one of the primary constraints in ML training is the disparity in feature scales. Since algorithms often prioritize attributes with larger values, proper feature scaling is necessary to ensure balanced learning. Feature scaling also helps machine learning and deep learning algorithms train and converge faster, which improves model performance. There are multiple methods for scaling data, but this study utilizes normalization as it is better suited for non-gaussian data distributions.

Normalization works by adjusting numerical values in a dataset to a consistent range, usually between 0 and 1 or sometimes –1 and 1, to maintain uniformity across features. This method is particularly helpful when features are on vastly different scales and lack extreme values. Normalization effectively scales data to a consistent range by using Equation 2


$$\begin{array}{l} X_{\text{new}}=\frac{X-X_{\text{min}}}{X_{\text{max}}-X_{\text{min}}} \end{array}$$ (2)

where:

- *X* is the original data value,
- *X*_min_ and *X*_max_ are the minimum and maximum values of the data feature, respectively.

This approach compresses data into an *n*-dimensional unit hypercube *Scikit-Learn* offers a transformer called MinMaxScaler for this process, which was utilized in this study.

### 2.5 The proposed CONN architecture

**Figure 9:**
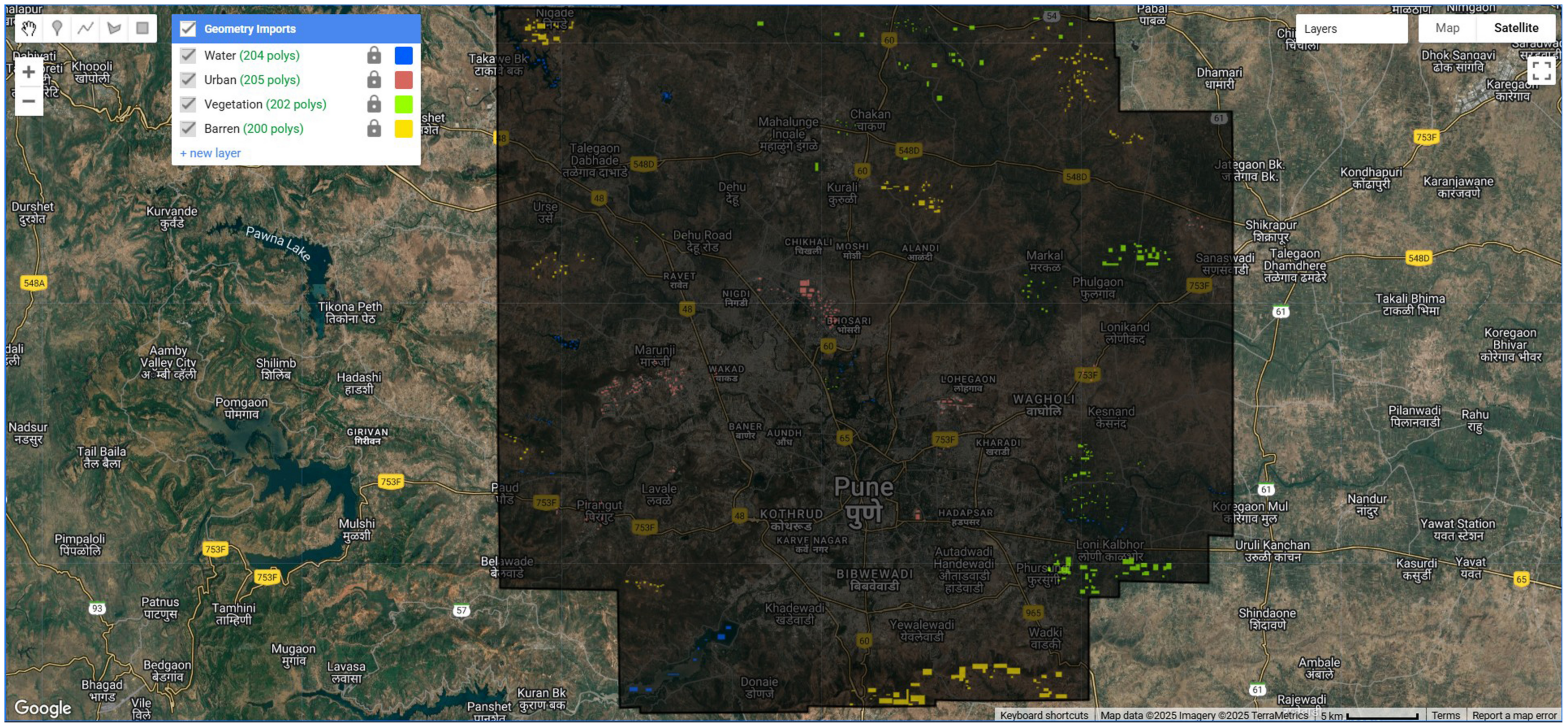
Training and test sample collection using polygon tool in GEE.

**Figure 10:**
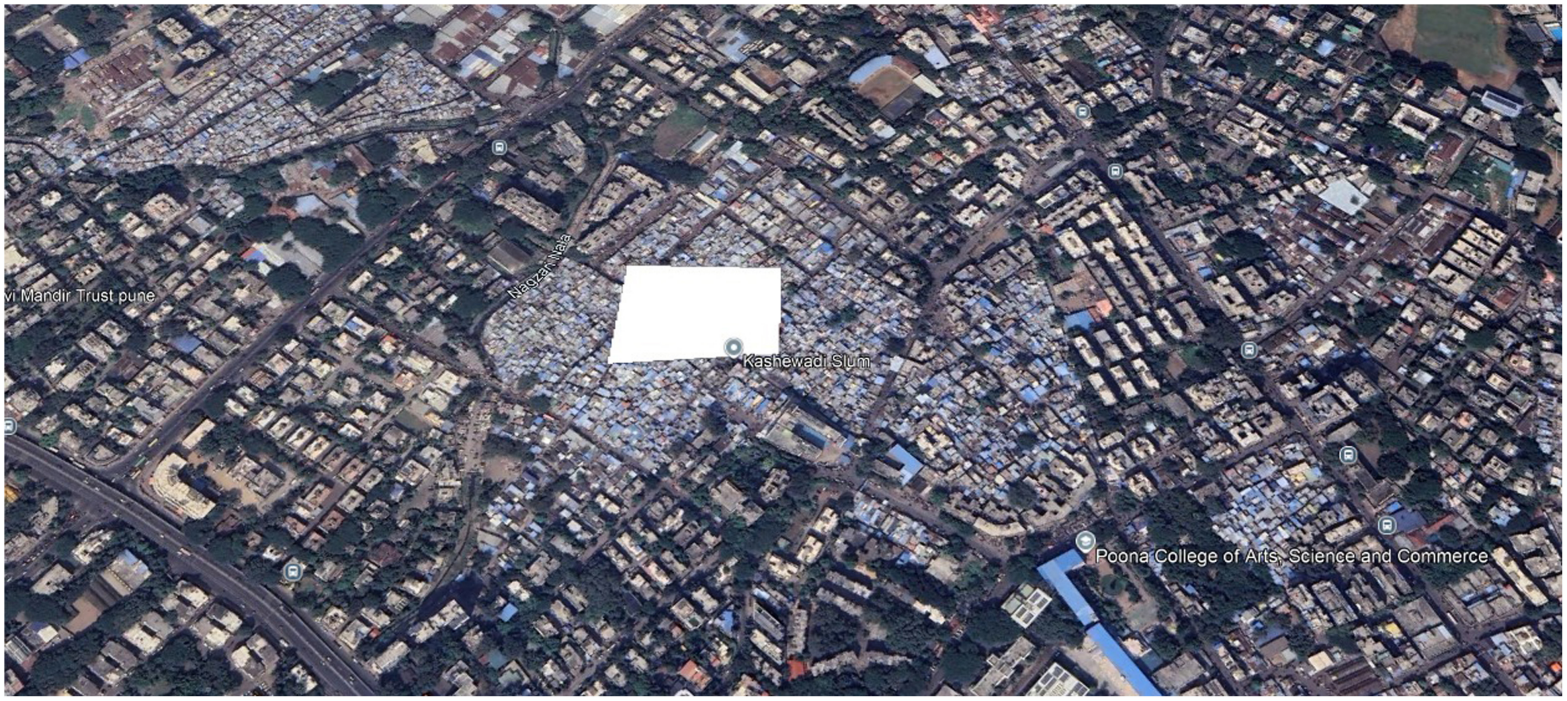
Ground truth sample.

**Figure 11:**
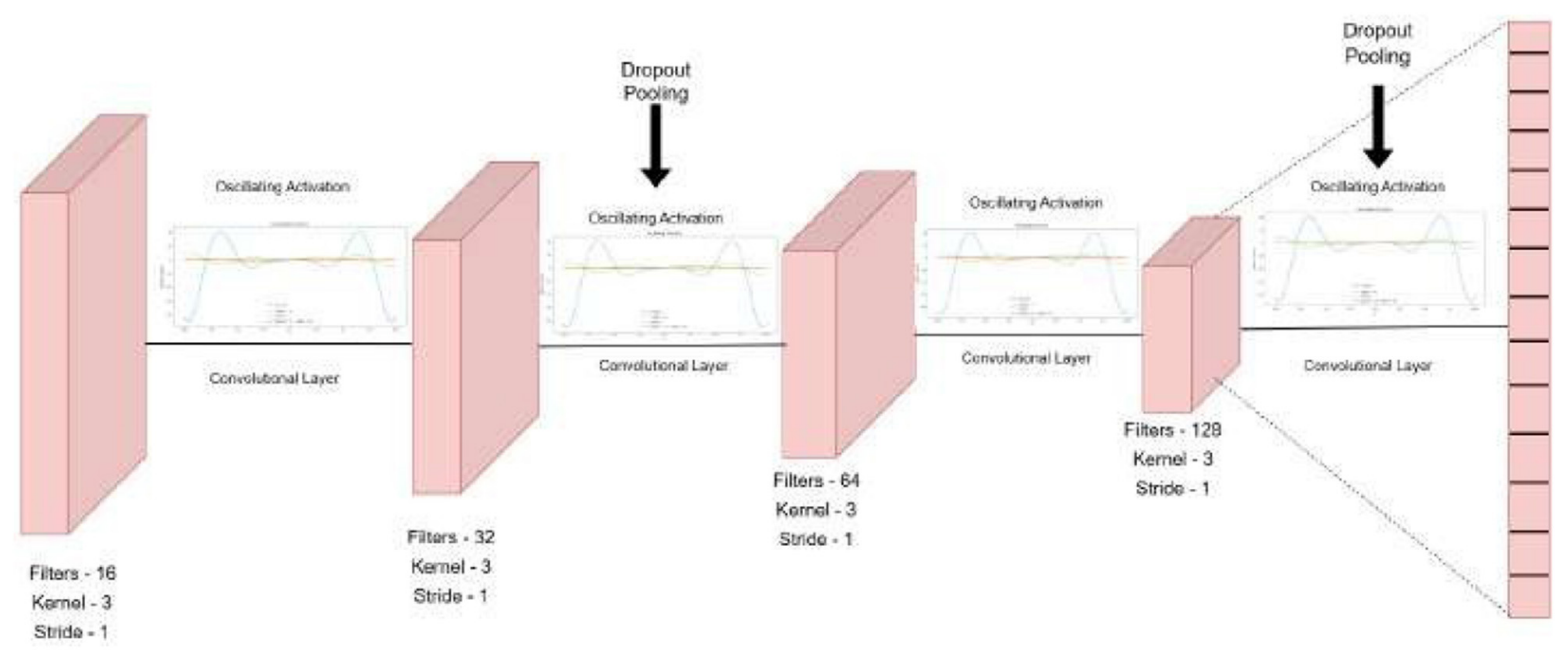
Oscillatory activated convolution layers.

The proposed CONN model is designed to process nineteen raster-based input features, with representative samples shown in Figure 9 and the corresponding ground truth illustrated in Figure 10. These inputs are organized into batches of 32 during training to ensure efficient learning and optimization. The overall architecture of the model is illustrated in Figure 11.

It gives the output classification probabilities for each of the four classes and classifies the region based on the given output probabilities. The sampling steps and the data are split into train and test batches of 80–20 ratio. Table 6 presents the detailed number of training and testing samples. The model is trained in mini-batches, and each epoch consists of forward and backward propagation steps through the network using the specified loss function. Model parameters are iteratively updated to minimize the loss, with the training process monitored using a separate validation (test) set to ensure generalization and avoid overfitting. This protocol facilitates reliable learning of the textural and spectral features present in the raster input data. It consists of stacked one-dimensional convolutional layers, coupled with a one-dimensional Max-Pooling layer for reducing the feature map size and efficient raster data analysis while extracting vital textural and spectral features from the raster data. The stacked CNN model gradually increases the output channel of the data, expanding it to 128 channels, therefore capturing more complex patterns and features from the data. These 128 extracted features, serve as input to the fully connected layers, which reduce the dimensionality gradually, by a factor of two, ensuring that there is no information lost in the flow of representations from one layer to the other.

**Table 6:** Sample distribution by class.

**Class**	**Urban**	**Vegetation**	**Barren**	**Waterbody**
Training/testing	5,205/2,471	11,074/2,438	29,593/3,081	32,506/9,815
Total	7,676	13,512	32,674	42,321

To prevent overfitting and serve as a regularization parameter, every pooling and fully connected layer is followed by a Dropout layer (Huang et al., 2021) with a dropout rate of 20%. To ensure smooth gradient flow during backpropagation and to prevent saturation and stagnation during the training of the model, the architecture utilizes oscillating activation functions after each convolutional and feed-forward layer (Rumelhart et al., 1986). These functions and their advantages in the training process have been further discussed in Section 2.5.2

**Figure 12:**
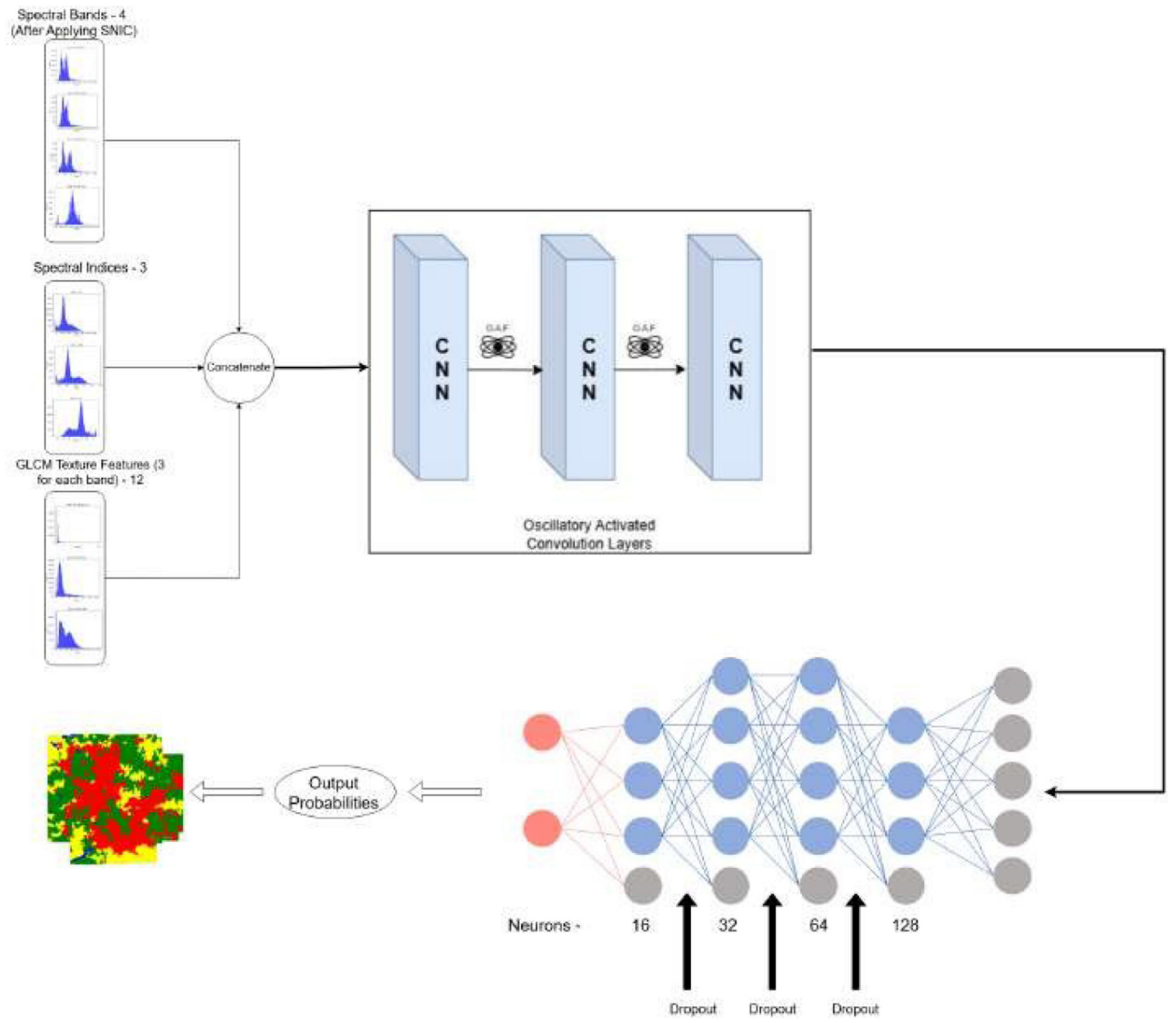
Oscillatory activated convolution layers.

In this study, we have chosen specific kernel sizes, strides, and filter counts for the CNN model to enhance feature extraction after thorough experimentation with a number of variations. The current parameters strike a balance between computational efficiency and the model's ability to identify significant patterns in the data. The architecture increases the number of filters after each layer by a factor of two, yielding a map of 128 channels before feeding into the decision head, which consists of a feed-forward neural network with dropout and oscillating activation functions in the end. The model architecture is shown in Figure 12.

#### 2.5.1 Convolutional and feed forward neural network

CNN architectures serve as the backbone of many image-classification and feature-extraction tasks today (Taye, 2023). By stacking multiple CNN layers, they are capable of learning local patterns within data, thereby extracting high-level spatial and texture features through learnable filters and pooling operations. This allows the network to build a hierarchical feature representation in the model's stored parameters as it progresses through each layer. The feed-forward neural networks in these cases serve as the decision head by classifying the learned features into their respective classes. Commonly employed activation functions like *ReLU, LeakyReLU*, and *Swish* suffers from commonly known issues in Deep Neural Networks like vanishing gradient and saturation problems (Hu et al., 2021). Recently discovered oscillatory activation functions show promising results due to their periodic nature and also their ability of possessing multiple hyperplanes as compared to commonly used activation functions, which only have one (Noel et al., 2025b). This property allows a single neuron to solve the XOR problem, thus demonstrating higher information retention capability.

#### 2.5.2 Oscillating activation functions

LULC classification using remote sensing data is highly non-linear due to the presence of overlapping spectral signatures, and the complex non-linear decision-making capability of oscillatory activation functions can be beneficial for providing accurate classification in such cases. These oscillatory activation functions, inspired by biological neurons from the neuro-cortex in a human brain, have a unique property of partitioning its input space with more than one hyperplane as compared to various state-of-the-art, non-linear activation functions like *ReLU, LeakyReLU*, and *Swish* as seen in Equations 3–5.

The ability to possess multiple decision boundaries significantly boosts the representative power of neurons incorporating these activation functions. Due to the enhanced ability of each neuron to hold complex representations, the overall ability of a neural network utilizing such neurons is also significantly increased. This, in turn, leads to an increase in training speed and efficiency. This can also be observed in the results of this study. It has also been observed that the proposed model performs and generalizes better with fewer neurons.


$$f(x)=\{\begin{array}{ll} x, & \text{if }x\geq 0\\ \alpha x, & \text{if }x<0 \end{array}$$ (3)

where α is a small constant (e.g., α = 0.01).


$$\begin{array}{l} f\left(x\right)=\max\left(0,x\right) \end{array}$$ (4)

$$\begin{array}{l} f\left(x\right)=x\cdot \sigma \left(\beta x\right)=\frac{x}{1+e^{-\beta x}} \end{array}$$ (5)

where σ(*x*) is the sigmoid function and β is a parameter (often set to 1).

**Figure 13:**
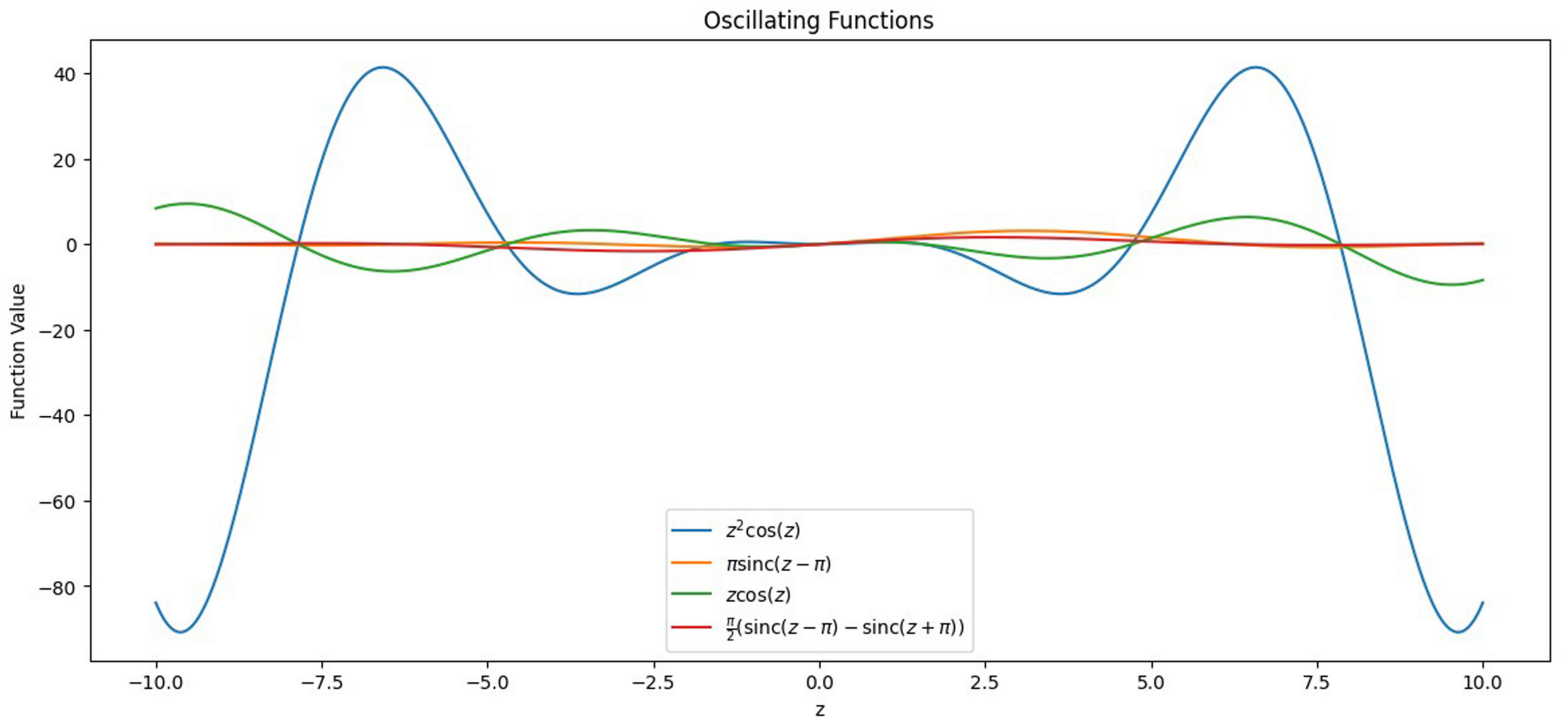
Plot of the oscillating activation functions incorporated in the study in the limits [–10, 10].

**Figure 14:**
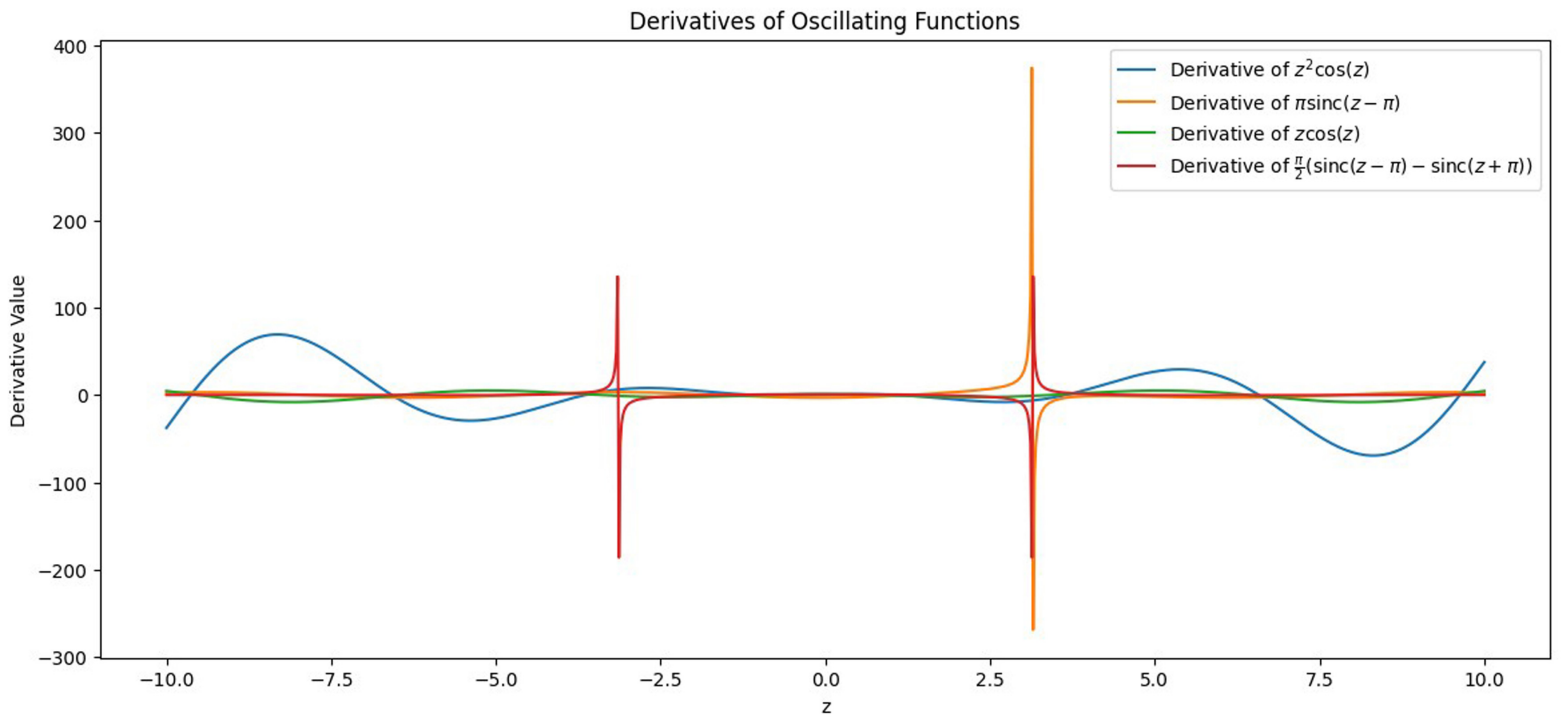
Plot of the derivatives of the oscillating activation functions incorporated in the study in the limits [–10, 10].

Their property of being differentiable at all points ensures that the neuron doesn't saturate and gives derivative values close to 0, which leads to the underflow problem while calculating the derivative of the loss function with respect to the model parameters. This is termed as vanishing gradients (Tan and Lim, 2019), a common problem during training of deep neural nets, and can be an encumbrance to the model's ability to learn from the data collection and store its representation in the form of weights. Saturation (Kolbusz, Janusz et al., 2018), on the other hand, is a stage where the output values of a neuron are close to the activation function's boundary values. It negatively affects both the information capacity and the learning ability of a neural network. The proposed model architecture utilizes four oscillating activation functions and gives a comprehensive analysis and comparison between each activation function along with the existing state-of-the-art non-linear activation functions such as *ReLU, LeakyReLU*, and *Swish*. The activation functions used in the study are *z*^2^*cos*(*z*), Shifted Sin Unit *(SSU)*, Growing Cosine Unit *(GCU)*, and Decaying Sin Unit *(DSU)* and are defined in Equations 6–9. The behavior of these functions and their derivatives is shown in Figures 13, 14.


$$\begin{array}{l} f\left(z\right)=z^{2}cos\left(z\right) \end{array}$$ (6)

$$\begin{array}{l} f\left(z\right)=z\cos\left(z\right) \end{array}$$ (7)

$$\begin{array}{l} f\left(z\right)=\pi \mathrm{sinc}\left(z-\pi \right) \end{array}$$ (8)

$$\begin{array}{l} f\left(z\right)=\frac{\pi }{2}\left(\mathrm{sinc}\left(z-\pi \right)-\mathrm{sinc}\left(z+\pi \right)\right) \end{array}$$ (9)

Equations 8, 9 can be written as:


$$\begin{array}{l} f\left(z\right)=\pi \frac{\sin\left(z-\pi \right)}{z-\pi } \end{array}$$ (10)

$$\begin{array}{l} f\left(z\right)=\frac{\pi }{2}\left(\frac{\sin\left(z-\pi \right)}{z-\pi }-\frac{\sin\left(z+\pi \right)}{z+\pi }\right) \end{array}$$ (11)

#### 2.5.3 Loss functions

The model applies the weighted cross-entropy loss function on the outputs of the softmax layer. The usage of an additional parameter, weight *w*_*i*_ in the function, proves to be vital while handling datasets with uneven class distributions, especially in the use-cases of LULC. In such instances, employing standard Cross-entropy loss can bias the model toward the majority class, often resulting in misclassifications and reduced performance on minority classes during test data inferencing (Ebrahimy et al., 2022). Weighted Cross-Entropy Loss (in Equation 12) addresses this issue by allocating distinct weights to each class, thereby equilibrating their impact throughout training. The class weights are determined according to the frequency with which each label appears in the dataset Equation 13 and depend inversely on it. For the optimization procedure for each epoch, utilize the state-of-the-art, adaptive optimization algorithm, the AMS-Grad variant of the Adam (Kingma and Ba, 2017) optimizer. This trains the model and updates the parameters during backpropagation. The AMSGrad version has been proven to be better than the base version of Adam in terms of convergence and also improving the generalizability of the model (Reddi et al., 2019).


$$L=-\sum_{i=1}^{C}w_{i}\cdot y_{i}\log({\hat{y}}_{i})$$ (12)

$$\begin{array}{l} w_{i}=\frac{N}{n_{i}\times C} \end{array}$$ (13)

where *N* denotes the total number of samples, *n*_*i*_ represents the number of samples belonging to class *i*, *C* is the total number of classes, *L* refers to the weighted loss, *w*_*i*_ indicates the weight assigned to class *i*, *y*_*i*_ is the true label of class *i*, and ŷ_*i*_ denotes the predicted probability for class *i*. The end-to-end training of the model is done by backpropagation and weight updates as denoted in Equation 14. The model has been trained for a total of 100 epochs for each of the oscillating activation functions, in order to perform a thorough comparison between each of the activation functions. In addition to this, a comparison was also carried out among the various machine learning algorithms used for LULC.


$$\begin{array}{l} \theta_{ij}:=\theta_{ij}-\eta \frac{\partial E}{\partial \theta_{ij}} \end{array}$$ (14)

where: θ_*ij*_ represents the weight or parameter between neuron *i* and neuron *j*, η is the learning rate, *E* is the error (or loss) function, $\frac{\partial E}{\partial \theta_{ij}}$ is the partial derivative of the error with respect to θ_*ij*_. This formula updates θ_*ij*_ by moving it in the direction that reduces the error, with η controlling the step size of each update. The values of each of the hyper-parameters of the model have been listed in Table 7.

**Table 7:** Hyperparameters used for the proposed CONN model.

**Hyperparameter**	**Description**	**Value**
Batch size	Number of samples in each batch	32
Learning rate (η)	Controls the step size in weight updates	0.001
Dropout rate	Probability of setting a neuron to zero during training	0.2
Filters (feature maps)	Number of filters in each Conv1D layer	{16, 32, 64, 128}
Kernel size	Size of the filter for Conv1D layers	3
Stride	Step size for moving the kernel	1
Pooling size	Size of MaxPooling layer	2
Activation functions	Non-linear functions for each layer	Oscillatory, Softmax
Optimizer	Algorithm used to minimize loss	Adam (AMSGrad)
Loss function	Function used to calculate error	Weighted cross-entropy
Epochs	Number of times the model sees the entire dataset	100
Weight initialization	Method to initialize weights	Xavier initialization

### 2.6 Performance metrics

The metrics used to evaluate the performance of the CONN include accuracy score, precision, recall, F1-score, confusion matrix, user's accuracy, producer's accuracy, and Kappa score. This subsection aims to discuss the efficacy of each of the selected metrics in gauging certain aspects of the performance of the models being compared. Those metrics are presented in Table 8. where *TP* is true positives, *TN* is true negatives, *FP* is false positives, *FN* is false negatives, *P*_*o*_ is the observed agreement and *P*_*e*_ is the random agreemen. *b* and *c* are disagreeing cases where one model predicts positive and the other predicts negative in the McNemar test.

**Table 8:** Metrics and their formulas for classification evaluation.

**Metric**	**Definition**	**Formula**
Accuracy	Defined as the ratio of correctly classified data samples out of the total dataset.	$\text{Accuracy}=\frac{TP+TN}{TP+TN+FP+FN}$
Precision	Indicates the proportion of true positive results in all positive predictions in the dataset.	$\text{Precision}=\frac{TP}{TP+FP}$
Recall (producer's accuracy)	Also known as Sensitivity, measures correctly classified actual positives.	$\text{Recall}=\frac{TP}{TP+FN}$
F1-score	The harmonic mean of precision and recall.	$F1\;Score=2\cdot \frac{\text{Precision}\cdot \text{Recall}}{\text{Precision}+\text{Recall}}$
		
Kappa score	Measures the relation between the predicted and actual classifications, accounting for the agreement that could occur by chance.	$\kappa =\frac{P_{o}-P_{e}}{1-P_{e}}$
Agreement *P*_*o*_	The number of instances where predictions and actual classifications agree.	$P_{\text{o}}\text{=}\sum_{i\text{=1}}^{n}n_{ii}$
Random *P*_*e*_	The expected frequency of agreement due to chance for Kappa calculation.	$P_{e}=\sum_{i=1}^{n}\frac{n_{i+}\times n_{+i}}{N}$
McNemar test (Chi-square)	Statistical test to compare differences on paired nominal data (classifier outputs).	$\chi^{2}=\frac{{\left(b-c\right)}^{2}}{b+c}$
Exact *p*-value	Probability value associated with McNemar test.	$p=2\sum_{i=0}^{\min(b,c)}\left(\begin{array}{c} b+c\\ i \end{array}\right)0.5^{b+c}$

### 2.7 Software and hardware specifications

The dataset for Landsat-8 SR was obtained from GEE utilizing the JavaScript API version 0.1.397, it was also utilized to implement algorithms such as SNIC and GLCM. For data preprocessing, model training and experimentation, Python version 3.10 was used, along with the PyTorch framework version 2.0. The hardware employed for training the model is an Intel Xeon CPU (2.3 GHz), 16GB of DDR4 RAM, and 12GB of VRAM.

## 3 Results

The experimental setup for the study is presented in Sections 2.5, 2.7 and Table 9. CONN comprises four layers of Convolutional Oscillatory activated layers with a kernel size of three and a stride of 1, along with two dropout and max pooling layers of kernel size 2. This architecture is then followed by Feed feed-forward oscillatory Neural Network of four hidden layers and each followed by a dropout layer.

**Table 9:** Layer configuration details of the employed CONN architecture along with oscillatory activation functions.

**Layer name**	**Output dimensions**	**non-linearity**
Convolutional layer-1	[–1, 16, 17]	Oscillatory activation
Convolutional layer-2	[–1, 32, 15]	Oscillatory activation
Max pooling-3	[–1, 32, 7]	None
Dropout-4	[–1, 32, 7]	None
Convolutional layer-5	[–1, 64, 5]	Oscillatory activation
Convolutional layer-6	[–1, 128, 3]	Oscillatory activation
Max pooling-7	[–1, 128, 1]	None
Dropout-8	[–1, 128, 1]	None
Feed forward neural network-9	[–1, 64]	Oscillatory activation
Dropout-10	[–1, 64]	None
Feed forward neural network-11	[–1, 32]	Oscillatory activation
Dropout-12	[–1, 32]	None
Feed forward neural network-11	[–1, 16]	Oscillatory activation
Dropout-12	[–1, 16]	None
Feed forward neural network-13	[–1, 4]	Softmax

The results presented in Table 10 indicate that the CONN model outperforms the conventionally activated functions across all the chosen metrics. There has been an especially large improvement when it comes to the Kappa score with DSU being nearly 0.4 higher than Swish. The confusion matrix for the proposed CONN-DSU is present in Table 11. This indicates the higher reliability of the CONN model over conventionally activated models when it comes to imbalanced datasets such as remote sensing data. Additionally, the CONN model improves greatly upon the precision for each class. This indicates that the CONN model makes significantly fewer misclassifications, even in cases where classes have very similar spectral characteristics, such as the barren and urban classes.

**Table 10:** Comparison of activation functions based on performance metrics.

**Activation function**	**Train-ing Acc**.	**Test Acc**.	**Pre-cision**	**Re-call**	**F1-Score**	**Kappa Score**	**User Acc. Class 1**	**User Acc. Class 2**	**User Acc. Class 3**	**User Acc. Class 4**
ReLU	95.251	93.185	93.138	93.185	93.081	90.636	95.948	90.894	94.383	91.808
LeakyReLU	96.102	93.167	94.098	93.367	93.440	91.204	98.231	92.330	95.495	91.980
Swish	96.259	93.782	93.023	93.782	93.814	91.824	99.191	91.058	95.760	90.663
***z*^2^cos(*z*)**	**98.764**	**95.091**	**95.480**	**95.091**	**95.143**	**92.362**	**99.591**	**97.293**	**98.141**	**94.715**
**SSU**	**99.647**	**95.126**	**95.156**	**95.726**	**95.769**	**94.344**	**99.474**	**98.195**	**97.631**	**94.571**
**GCU**	**99.998**	**95.327**	**95.826**	**95.327**	**95.420**	**93.180**	**99.352**	**94.299**	**98.385**	**94.051**
**DSU**	**99.999**	**95.979**	**95.570**	**95.979**	**95.082**	**95.199**	**99.291**	**96.637**	**98.663**	**94.685**

Values in bold denote the highest accuracy.

**Table 11:** Confusion matrix for proposed CONN-DSU.

**Classified data**	**Reference data**					**Precision**
	**Class 0**	**Class 1**	**Class 2**	**Class 3**	**Total**	
Urban (0)	2,402	15	11	43	2,471	0.97
Vegetation (1)	14	2,348	25	51	2,438	0.96
Barren (2)	13	11	3,016	41	3,081	0.98
Waterbody (3)	93	67	332	9,323	9,815	0.95
Total	2,522	2441	3,384	9,458	17,805	–
Recall	0.95	0.96	0.89	0.99	–	–
**Overall accuracy: 95.79%**						
Random	350.00	334.24	585.57	5,213.71	–	–
Agreement	2402	2348	3016	9,323	–	–
**Kappa: 95.19%**						
**F1 score: 95.08%**						

The color shading represents the magnitude of the cell values; darker red cells indicate higher counts of true positives, while lighter red cells indicate comparatively lower counts of true positives.

**Figure 15:**
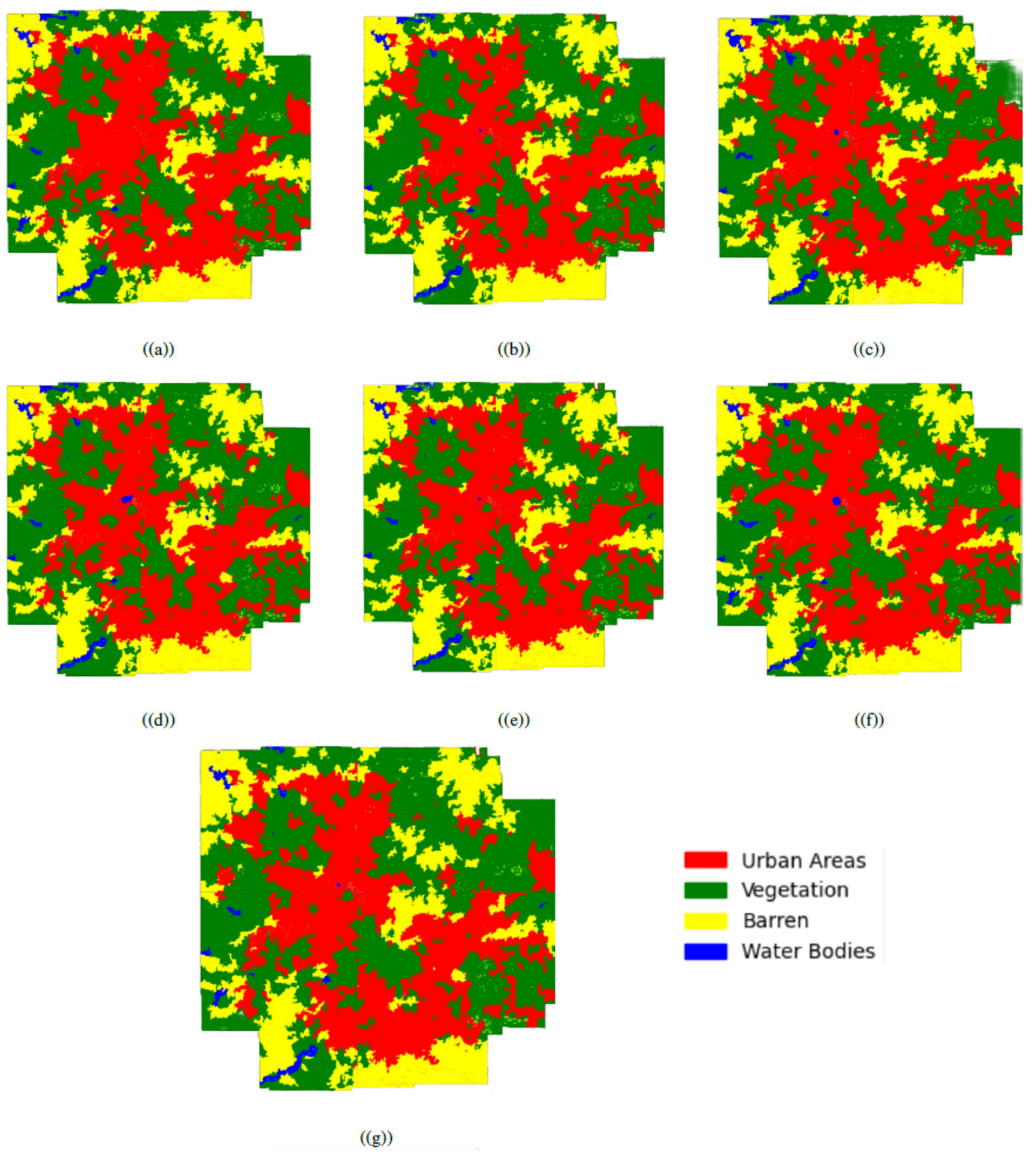
The classification results for the dataset using the following activation functions, **(a)** ReLU, **(b)** LeakyReLU, **(c)** Swish, **(d)** *z*^2^ cos(z), **(e)** DSU, **(f)** GCU, and **(g)** SSU, respectively.

**Figure 16:**
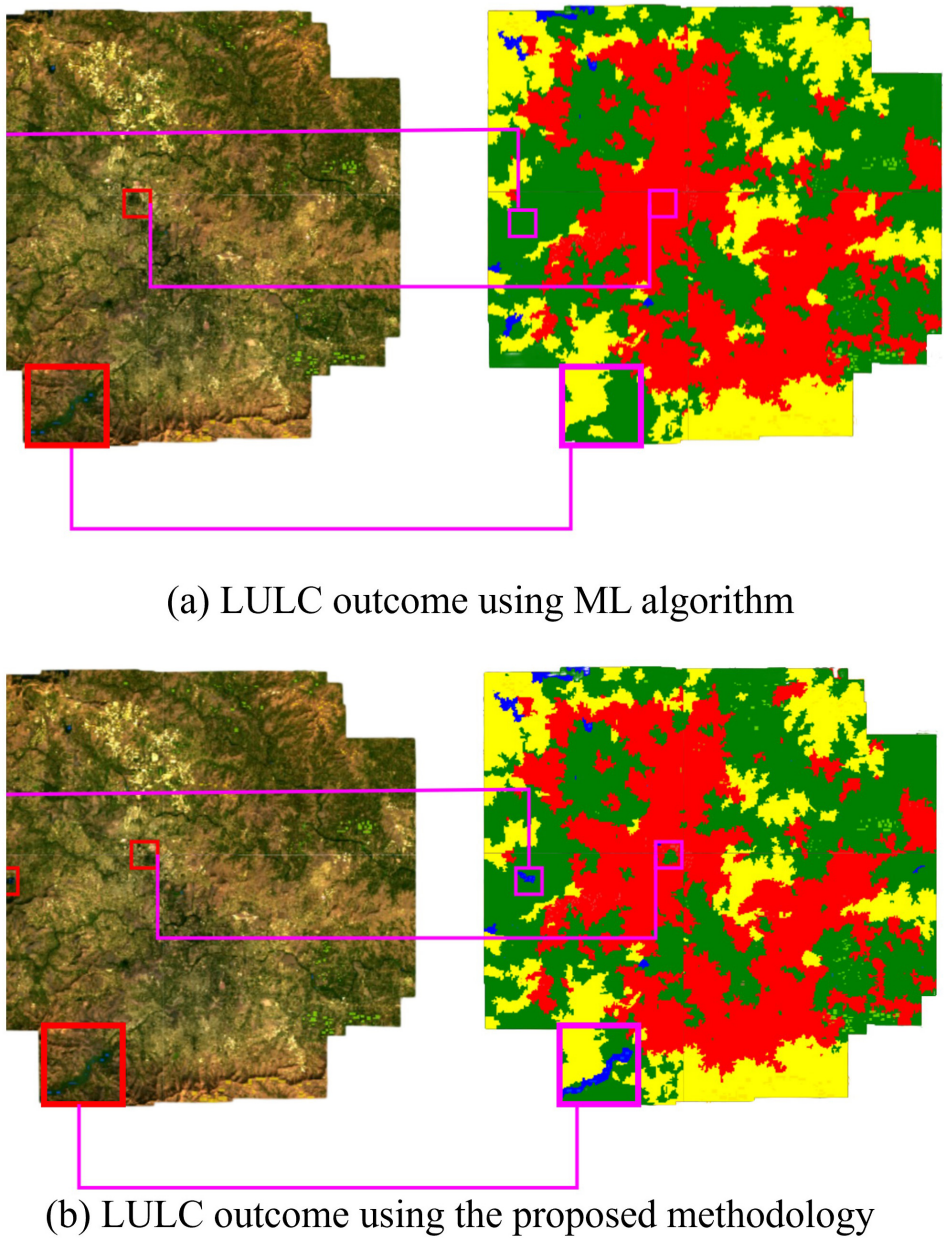
**(a)** LULC outcome using the proposed methodology and **(b)** LULC outcome of the ML Techniques.

**Figure 17:**
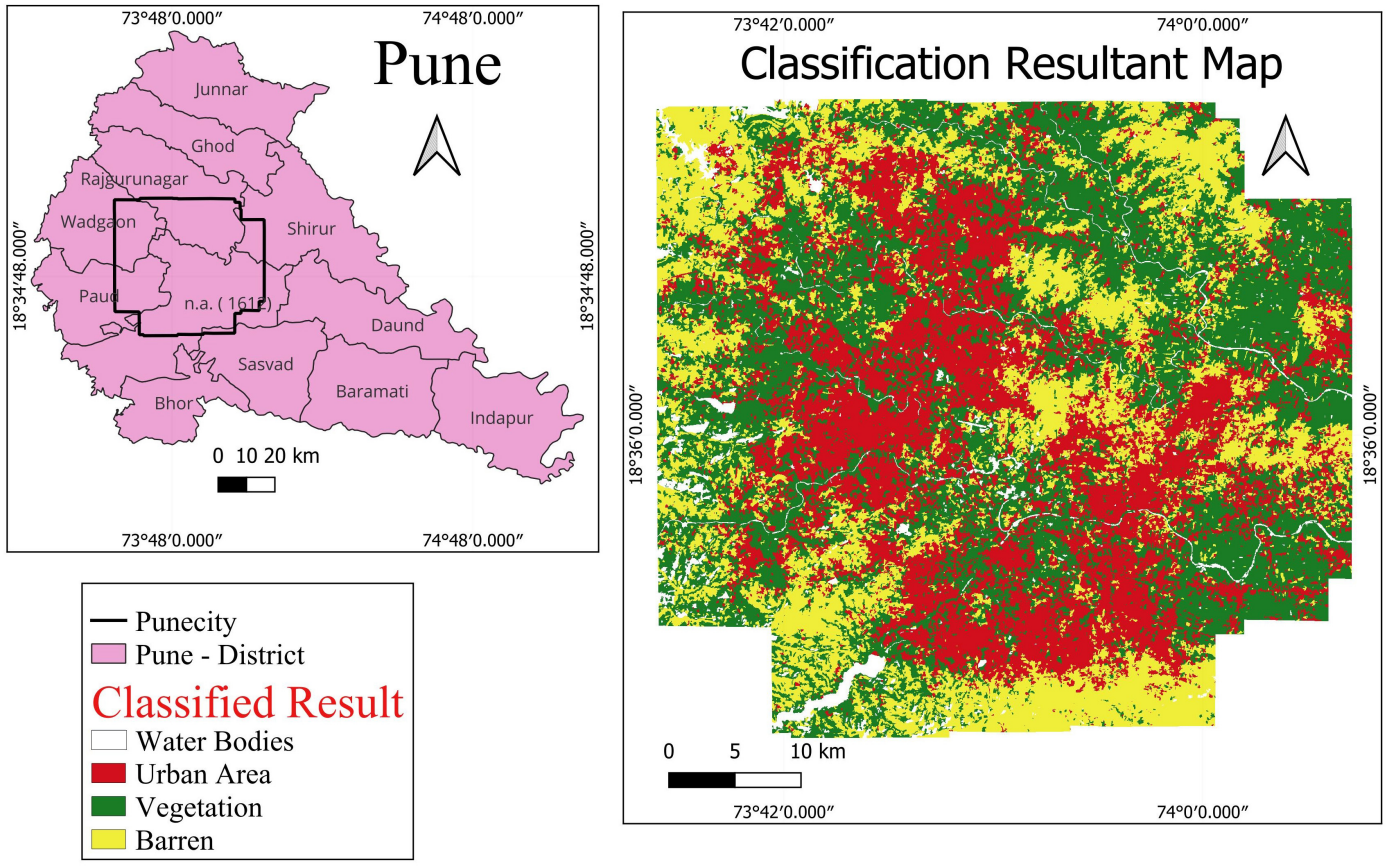
LULC maps of the study area using proposed CONN-DSU. Note that we use QGIS software(https://qgis.org/en/site/, version: 3.24.2) for plotting.

The ability of the oscillatory functions to be able to represent more information with the same number of neurons allows the CONN model to build a more complex representation of the classes, deterring over-reliance on spectral features alone for classification. This ability of the CONN model is also showcased in Figure 15, with the CONN model being able to classify smaller objects of some classes more accurately as we can see with the water body and vegetation patch in Figure 16. The spatial classification output map of the current study area is presented Figure 17.

**Figure 18:**
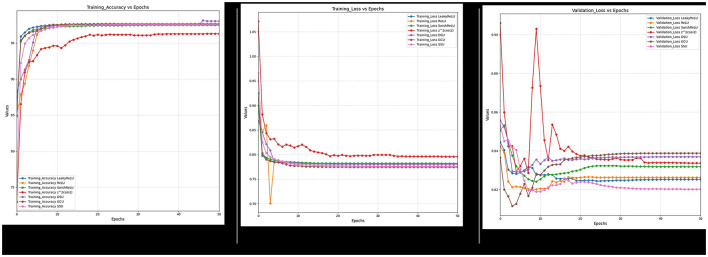
Ablated data training accuracy, loss, and validation loss for spectral feature.

**Figure 19:**
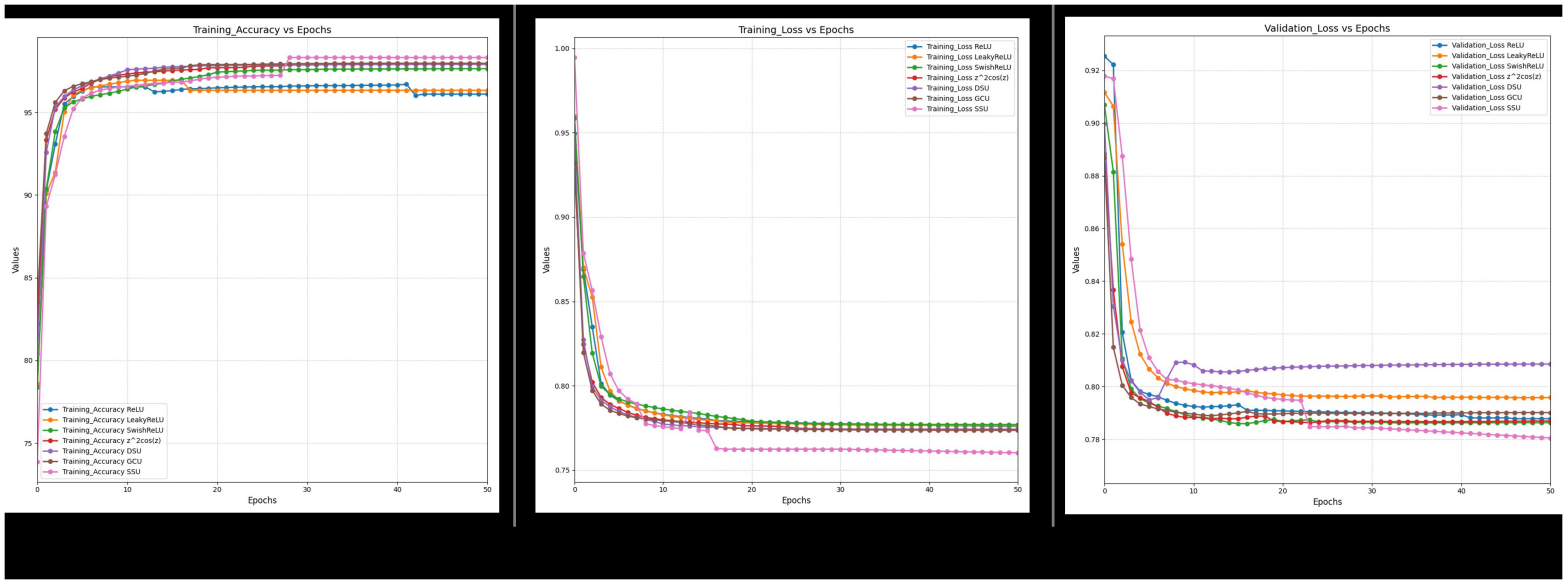
Ablated data training accuracy, loss, and validation loss for spectral and indices feature.

**Figure 20:**
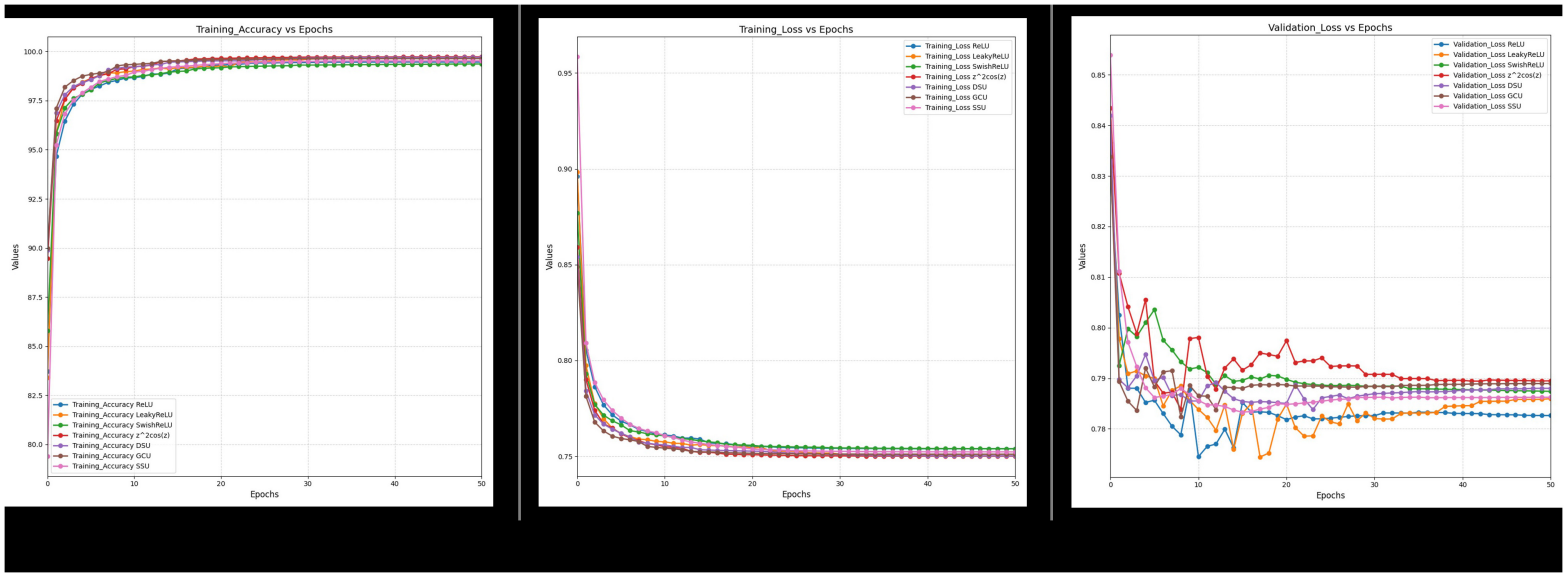
Ablated data training accuracy, loss, and validation loss for Spectral and texture feature.

**Figure 21:**
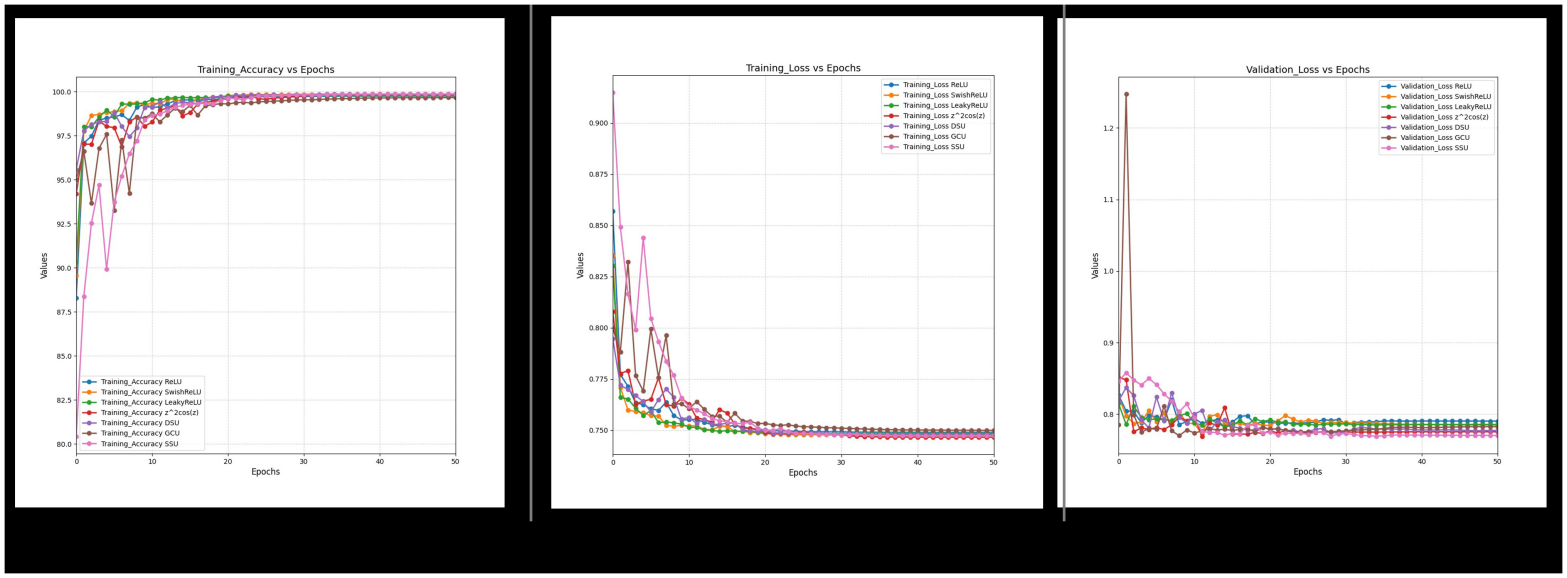
Training accuracy, loss and validation loss for non-ablated data.

Furthermore, it can also be observed from Figures 18–21 that over a period of 50 epochs, the proposed CONN model equipped with oscillatory activation functions, such as SSU, converge much faster than conventional activation functions. In addition to the comparison of the activation functions over the entire dataset, this study also performs an ablation study which tests the robustness of the CONN model as compared to conventionally activated CNNs when some features are not present. The results of the ablation study are presented in the following section.

## 4 Discussion

This paper performs an ablation study by removing parts of the feature set used in the original dataset and comparing the performance of both ML and DL models for these variations. The variations utilized are *Spectral features, spectral and textural features*, and *Spectral features and spectral indices*. The compared DL models include the proposed CONN model along with CNN models using ReLU, LeakyReLU, and Swish activation functions. The ML models chosen for this ablation study are Random Forest (RF), Support Vector Machine (SVM), Naive Bayes (NB), and AdaBoost. Additionally, the hyperparameters of these ML models were optimized using GridSearchCV, and the hyperparameters utilized for the test can be seen in Table 12. This test was performed in order to ascertain the importance of each feature, in addition to the CONN model's ability to work with fewer features. When utilizing remote sensing data, obtaining reliable data for all features may not be feasible. In such cases, the model should also possess the capability to train on suboptimal data and still provide serviceable results. Additionally, this test is also beneficial in identifying the importance of individual features.

**Table 12:** Hyperparameters utilized for the Listed ML models in the study.

**Model**	**Hyperparameters**
RF	n_estimators: 200, random_state: 42
SVM	C: 0.1, gamma: 'scale', kernel: 'linear'
AdaBoost	algorithm: 'SAMME', learning_rate: 0.1, n_estimators: 200

The results of the study for all the DL models, as shown in Table 13, indicate that for all three variations of the dataset, the CONN model utilizing oscillatory activation functions outperforms all the conventionally activated DL models for all the chosen metrics. Furthermore, it can be observed in Figure 21 that despite the additional performance, the CONN model provides similar convergence speeds to conventional activation functions and, in some cases, shows sustained improvements till much higher epochs. Additionally, the results for ML models presented in Tables 14, 15 further establish the better performance of the CONN model over ML models on all three variations of the dataset.

**Table 13:** Results with training accuracy and other metrics.

**Data type**	**Activation function**	**Train-ing Acc**.	**Test Acc**.	**Pre-cision**	**Re-call**	**F1-Score**	**Kappa score**	**User Acc. Class 1**	**User Acc. Class 2**	**User Acc. Class 3**	**User Acc. Class 4**
Spectral features	ReLU	97.84	91.59	91.40	91.59	91.73	87.96	88.72	88.20	98.80	90.21
	LReLU	97.53	91.01	91.67	91.01	90.62	87.13	88.31	88.85	98.80	92.17
	Swish	97.47	90.53	92.04	90.53	90.74	85.54	89.31	88.85	98.80	92.17
	***z*^2^cos(*z*)**	**96.28**	**93.28**	**93.98**	**93.28**	**93.35**	**90.42**	**93.72**	**90.76**	**98.89**	**95.90**
	**SSU**	**97.58**	**95.68**	**94.38**	**93.68**	**93.79**	**90.09**	**92.51**	**85.43**	**99.41**	**94.22**
	**GCU**	**97.65**	**95.90**	**95.28**	**95.90**	**95.35**	**91.09**	**94.57**	**84.53**	**99.37**	**94.90**
	**DSU**	**98.22**	**95.78**	**95.48**	**95.28**	**95.50**	**91.00**	**94.19**	**86.38**	**99.80**	**95.16**
Spectral features and textural	ReLU	99.45	91.31	91.91	91.31	91.40	88.18	94.26	93.29	96.01	91.72
	LReLU	99.69	92.54	92.19	92.54	92.65	89.99	95.78	94.18	96.87	90.83
	Swish	99.43	92.21	92.95	92.21	92.32	89.05	95.26	95.47	96.12	91.09
	***z*^2^cos(*z*)**	**99.73**	**95.92**	**95.75**	**95.92**	**95.02**	**92.12**	**98.54**	**97.21**	**99.62**	**93.28**
	**SSU**	**99.52**	**96.97**	**96.61**	**96.97**	**96.08**	**93.67**	**97.41**	**97.82**	**99.39**	**94.40**
	**GCU**	**99.63**	**95.51**	**94.26**	**95.51**	**95.62**	**92.99**	**97.93**	**95.89**	**99.82**	**93.46**
	**DSU**	**99.69**	**95.41**	**94.48**	**95.41**	**95.58**	**92.85**	**98.30**	**96.34**	**99.79**	**92.77**
Spectral features and indices	ReLU	96.33	89.07	90.25	89.07	90.11	89.82	92.98	94.50	94.37	95.01
	LReLU	96.32	89.57	90.95	89.57	90.66	91.53	92.46	94.38	94.17	93.98
	Swish	97.63	91.25	91.14	91.25	91.29	91.10	91.18	94.75	93.98	95.36
	***z*^2^cos(*z*)**	**97.91**	**95.87**	**96.05**	**95.87**	**95.92**	**94.50**	**95.67**	**95.70**	**95.13**	**96.45**
	**SSU**	**98.30**	**94.50**	**94.84**	**94.50**	**94.58**	**94.40**	**96.35**	**95.83**	**94.95**	**95.75**
	**GCU**	**97.95**	**95.33**	**95.50**	**95.33**	**95.38**	**93.66**	**95.99**	**94.76**	**95.16**	**95.60**
	**DSU**	**97.89**	**95.78**	**95.69**	**95.78**	**95.72**	**94.47**	**96.66**	**95.55**	**95.94**	**95.58**
Spectral features, indices and textural features	ReLU	95.25	93.18	93.13	93.18	93.08	90.63	95.94	90.89	94.38	91.80
	LReLU	96.10	93.16	94.09	93.36	93.44	91.20	98.23	92.33	95.49	91.98
	Swish	96.25	93.78	93.02	93.78	93.81	91.82	99.19	91.05	95.76	90.66
	***z*^2^cos(*z*)**	**98.76**	**95.09**	**95.48**	**95.09**	**95.14**	**92.36**	**99.59**	**97.29**	**98.14**	**94.71**
	**SSU**	**99.64**	**95.12**	**95.15**	**95.72**	**95.76**	**94.34**	**99.47**	**98.19**	**97.63**	**94.57**
	**GCU**	**99.99**	**95.32**	**95.82**	**95.32**	**95.42**	**93.18**	**99.35**	**94.29**	**98.38**	**94.05**
	**DSU**	**99.99**	**95.97**	**95.57**	**95.97**	**95.08**	**95.19**	**99.29**	**96.63**	**98.66**	**94.68**

Values in bold denote the highest accuracy.

**Table 14:** Comparison of CONN (DSU) and ML models results on test data.

**Category**	**Model**	**Test accuracy**	**Test precision**	**Test recall**	**Test F1-score**
Spectral features	Random forest	93.540%	0.9431	0.9354	0.9370
	SVM	94.440%	0.9502	0.9444	0.9455
	Naive Bayes	81.470%	0.8120	0.8147	0.8053
	AdaBoost	80.380%	0.7034	0.8038	0.7460
	**CONN(DSU)**	**95.783%**	**0.9549**	**0.9528**	**0.9551**
Spectral features and indices	Random Forest	94.520%	0.9496	0.9452	0.9462
	SVM	94.670%	0.9519	0.9467	0.9477
	Naive Bayes	85.440%	0.8476	0.8544	0.8470
	AdaBoost	68.870%	0.5213	0.6887	0.5876
	**CONN(DSU)**	**95.789%**	**0.9570**	**0.9579**	**0.9572**
Spectral features and textural	Random Forest	91.140%	0.9187	0.9114	0.9113
	SVM	94.430%	0.9515	0.9443	0.9455
	Naive Bayes	87.580%	0.8825	0.8758	0.8765
	AdaBoost	88.980%	0.9070	0.8898	0.8920
	**CONN(DSU)**	**95.417%**	**0.9448**	**0.9542**	**0.9559**

Values in bold denote the highest accuracy.

**Table 15:** Training results for ML models on the ablated datasets.

**Category**	**Model**	**Train accuracy**	**Train precision**	**Train recall**	**Train F1-score**
Spectral features	Random forest	100.000%	1.0000	1.0000	1.0000
	SVM	96.320%	0.9606	0.9600	0.9612
	Naive Bayes	84.242%	0.8419	0.8424	0.8365
	AdaBoost	80.470%	0.8109	0.8047	0.8075
Spectral features and indices	Random forest	100.000%	1.0000	1.0000	1.0000
	SVM	95.100%	0.9518	0.9512	0.9502
	Naive Bayes	87.537%	0.8794	0.8753	0.8735
	AdaBoost	74.201%	0.5895	0.7420	0.6566
Spectral features and textural	Random forest	100.000%	1.0000	1.0000	1.0000
	SVM	96.162%	0.9616	0.9616	0.9620
	Naive Bayes	94.191%	0.9426	0.9419	0.9421
	AdaBoost	92.290%	0.9235	0.9229	0.9126

### 4.1 Statistical analysis of the performance

The McNemar test was conducted to determine whether there was a statistically significant difference in the classification errors (discordant cases) between the proposed and the existing models, based on the confusion matrices provided. Table 16 presents several key parameters to assess the statistical significance of differences between models. These include the contingency values (*a*, *b*, *c*, *d*), the actual *p*-value, the significance threshold (α = 0.05), the observed chi-square (χ^2^) value, and the 95% confidence interval for this difference. In contingency value, *a* represents positive predictions, and *d* represents negative predictions by both models respectively, while *b* and *c* represent disagreeing cases where one model predicts positive and the other predicts negative. The null hypothesis H0 was taken to be: “there is no significant difference in the number of discordant pairs between the models.” Alternative hypothesis H1 was taken to be: “there is a significant difference in the number of discordant pairs between the models.” The statistical McNemar test was applied to compare the proposed DSU classification model with other DL models namely DL with ReLu, Leaky ReLu, Swish, z2 cos, GCU and SSU and Random Forest, SVM, Na'´ıve Bayes, and Adaboost ML models. All comparisons resulted in extremely low *p*-values, consistently below the conventional threshold (α = 0.05), confirming that the observed differences are statistically significant and not due to random variation. The observed chi-square values for most models were above the critical value of 3.841, indicating a clear improvement in classification performance by the proposed DL model with DSU activation function. Confidence intervals for these differences were consistently narrow, supporting the reliability of the findings. Even in the case of Random Forest model, the smallest difference noted remained significant. Moreover, the extremely large difference with Adaboost model highlights not only statistical but also substantial practical improvement. The statistical McNemar test shows that the DSU model's improvements are reliable and consistent, clearly supporting its better performance compared to other models.

**Table 16:** Statistical comparison: proposed DSU vs. other models.

**Models**	**Activation function**	***a***	***b***	***c***	***d***	**Actual *p*-value**	***p* Value**	**Observed χ^2^**	**Mean of interval**	**95% confidence interval**
DL	ReLU	16267	56	822	660	1.71 × 10^−175^	< 0.001	668.30	0.0430	0.0398–0.0462
	Leaky ReLU	16276	57	813	659	3.69 × 10^−172^	< 0.001	657.00	0.0425	0.0393–0.0456
	Swish	16118	200	1170	317	4.53 × 10^−151^	< 0.001	685.37	0.0545	0.0505–0.0585
	(Z^2^ Cos)	16267	47	822	669	7.99 × 10^−184^	< 0.001	691.16	0.0435	0.0403–0.0467
	GCU	16259	47	830	669	1.16 × 10^−153^	< 0.001	699.10	0.0440	0.0408–0.0472
	SSU	16247	54	842	662	9.13 × 10^−183^	< 0.001	693.00	0.0443	0.0410–0.0475
ML	RF	16780	49	309	667	2.93 × 10^−47^	< 0.001	188.30	0.0146	0.0125–0.0167
	SVM	16789	67	300	649	2.58 × 10^−36^	< 0.001	147.93	0.0131	0.0110–0.0152
	NB	15203	9	1886	707	0.0 Extremely	< 0.001	1859	0.1054	0.1009–0.1100
	Adaboost	12309	38	4780	678	0.0 Extremely	< 0.001	4665	0.2663	0.2729–0.2598

## 5 Conclusion and future works

This study proposes a convoluted oscillatory neural networks (CONN) architecture for accurate object based LULC classification. The proposed model utilizes the periodic, non-monotonic nature of the oscillatory activation functions with the deep, convoluted architecture of CNNs to accurately map and classify LULC. The study uses the surface reflectance images captured using Landsat 8 in the Pune area. GLCM textural features and spectral indices were then extracted from the objects segmented using the SNIC segmentation algorithm. According to experimental results, the proposed CONN with Decaying Sine Unit achieved an overall train accuracy of 99.999%, test accuracy of 95.979%, precision of 95.570%, recall of 95.979%, F1-score of 95.082%, and kappa score of 95.199%. The efficient gradient flow during Back propagation while training the proposed CONN allows the model to mitigate the vanishing gradient problem and to create a complex representation of data. The comparative analysis and comprehensive ablation study also highlight the superior performance of CONN over traditional ML approaches and conventional DL model-based architectures in the absence of critical features such as textures and spectral indices. It was also observed that although traditional ML models outperformed in training accuracy, they lack generalizing ability during testing, as showcased on the ablated test dataset. Since the neurons enhanced with oscillating activation functions have higher representation power, the number of neurons in the architecture has to be reduced in order to avoid overfitting. Future work can also explore transformer-based models, Quadratic Neural Networks (QNNs) (Noel and Muthiah-Nakarajan, 2024), along with time-series analysis. Transformer models have been proven to be better than traditional ANN-based architectures at capturing the correlation and difference in the time-series data (Nayak et al., 2024) and graph neural networks to further optimize classification performance and computational efficiency (Kavran et al., 2023).

## Data Availability

Publicly available datasets were analyzed in this study. This data can be found at: https://www.usgs.gov/.

## References

[B1] Achanta R. Shaji A. Smith K. Lucchi A. Fua P. S'´usstrunk S. . (2012). Slic superpixels compared to state-of-the-art superpixel methods. IEEE Trans. Pattern Anal. Mach. Intell. 34, 2274–2282. doi: 10.1109/TPAMI.2012.120 22641706

[B2] Achanta R. S'´usstrunk S. (2017). “Superpixels and polygons using simple non-iterative clustering,” in 2017 IEEE Conference on Computer Vision and Pattern Recognition (CVPR) (Honolulu, HI: IEEE), 4895–4904. doi: 10.1109/CVPR.2017.520

[B3] Amani M. Ghorbanian A. Ahmadi S. A. Kakooei M. Moghimi A. Mirmazloumi S. M. . (2020). Google earth engine cloud computing platform for remote sensing big data applications: a comprehensive review. IEEE. J. Sel. Top. Appl. Earth Obs. Remote Sens. 13, 5326–5350. doi: 10.1109/JSTARS.2020.3021052

[B4] Attri P. Chaudhry S. Sharma S. (2015). Remote sensing &GIS based approaches for lulc change detection-a review. Int. J. Curr. Eng. Technol. 5, 3126–3137.

[B5] Census of India (2024). Population Data of Pune Metropolitan Region. Office of the Registrar General & Census Commissioner, New Delhi, India.

[B6] Digra M. Dhir R. Sharma N. (2022). Land use land cover classification of remote sensing images based on the deep learning approaches: a statistical analysis and review. Arab. J. Geosci. 15. doi: 10.1007/s12517-022-10246-8

[B7] Dingle Robertson L. King D. J. (2011). Comparison of pixel-and object-based classification in land cover change mapping. Int. J. Remote Sens. 32, 1505–1529. doi: 10.1080/01431160903571791

[B8] Ebrahimy H. Mirbagheri B. Matkan A. A. Azadbakht M. (2022). Effectiveness of the integration of data balancing techniques and tree-based ensemble machine learning algorithms for spatially-explicit land cover accuracy prediction. Remote Sens. Appl. Soc. Environ. 27, 100785. doi: 10.1016/j.rsase.2022.100785

[B9] Fu W. Ma J. Chen P. Chen F. (2020). “Remote sensing satellites for digital earth,” in Manual of Digital Earth, eds. H. Guo, M. F. Goodchild, and A. Annoni (Cham: Springer), 55–123. doi: 10.1007/978-981-32-9915-3_3

[B10] Hansen M. C. Loveland T. R. (2012). A review of large area monitoring of land cover change using landsat data. Remote Sens. Environ. 122, 66–74. doi: 10.1016/j.rse.2011.08.024

[B11] Haralick R. M. Shanmugam K. Dinstein I. (1973). Textural features for image classification. IEEE Trans. Syst. Man Cyberne. 3, 610–621. doi: 10.1109/TSMC.1973.4309314

[B12] Hu Z. Zhang J. Ge Y. (2021). Handling vanishing gradient problem using artificial derivative. IEEE Access 9, 22371–22377. doi: 10.1109/ACCESS.2021.3054915

[B13] Huang S. Tang L. Hupy J. P. Wang Y. Shao G. (2021). Commentary review on the use of normalized difference vegetation index (NDVI) in the era of popular remote sensing. J. For. Res. 32:2719. doi: 10.1007/s11676-020-01176-w

[B14] Kamusoko C. (2017). “Importance of remote sensing and land change modeling for urbanization studies,” in Urban Development in Asia and Africa: Geospatial Analysis of Metropolises, eds. Y. Murayama, C. Kamusoko, A. Yamashita, and R. C. Estoque (Cham: Springer), 3–10. doi: 10.1007/978-981-10-3241-7_1

[B15] Kavran D. Mongus D. Žalik B. Lukač N. (2023). Graph neural network-based method of spatiotemporal land cover mapping using satellite imagery. Sensors 23:6648. doi: 10.3390/s23146648 37514942 PMC10384354

[B16] Kingma D. P. Ba J. (2017). Adam: A Method for Stochastic Optimization. doi: 10.48550/arXiv.1412.6980

[B17] Kolbusz J. Rozycki P. Lysenko O. Wilamowski B. M. (2018). “Neural networks saturation reduction,” in Artificial Intelligence and Soft Computing: 17th International Conference, ICAISC 2018, Zakopane, Poland, June 3-7, 2018, Proceedings, Part I 17 (Cham: Springer), 108–117. doi: 10.1007/978-3-319-91253-0_11

[B18] Kumari A. Karthikeyan S. (2023). Sentinel-2 data for land use/land cover mapping: a meta-analysis and review. SN Comput. Sci. 4:815. doi: 10.1007/s42979-023-02214-0

[B19] Maharashtra Government (2020). Geographical Coordinates of Pune. Government of Maharashtra, Mumbai, India.

[B20] Markham B. L. Barsi J. A. (2017). “Landsat-8 operational land imager on-orbit radiometric calibration,” in 2017 IEEE International Geoscience and Remote Sensing Symposium (IGARSS) (Fort Worth, TX: IEEE), 4205–4207. doi: 10.1109/IGARSS.2017.8127929

[B21] Nayak G. H. Alam M. W. Avinash G. Kumar R. R. Ray M. Barman S. . (2024). Transformer-based deep learning architecture for time series forecasting. Softw. Impacts 22:100716. doi: 10.1016/j.simpa.2024.100716

[B22] Nigar A. Li Y. Jat Baloch M. Y. Alrefaei A. F. Almutairi M. H. (2024). Comparison of machine and deep learning algorithms using google earth engine and python for land classifications. Front. Environ. Sci. 12:1378443. doi: 10.3389/fenvs.2024.1378443

[B23] Noel M. M. Arunkumar L. Trivedi A. Dutta P. (2025a). Oscillating activation functions can improve the performance of convolutional neural networks. Appl. Soft Comput. 175:113077. doi: 10.1016/j.asoc.2025.113077

[B24] Noel M. M. Bharadwaj S. Muthiah-Nakarajan V. Dutta P. D. G. B. A. (2025b). Biologically inspired oscillating activation functions can bridge the performance gap between biological and artificial neurons. Expert Syst. Appl. 266:126036. doi: 10.1016/j.eswa.2024.126036

[B25] Noel M. M. Muthiah-Nakarajan V. (2024). Efficient Vectorized Backpropagation Algorithms for Training Feedforward Networks Composed of Quadratic Neurons. doi: 10.48550/arXiv.2310.0290

[B26] Ouchra H. Belangour A. Erraissi A. (2022). A comparative study on pixel-based classification and object-oriented classification of satellite image. Int. J. Eng. Trends Technol. 70, 206–215. doi: 10.14445/22315381/IJETT-V70I8P221

[B27] Padigala B. (2012). Urbanization and changing green spaces in Indian cities (case study - city of Pune). Int. J. Geol. Earth Environ. Sci. 2, 148–156.

[B28] Pal R. Mukhopadhyay S. Chakraborty D. Suganthan P. N. (2023). A hybrid algorithm for urban lulc change detection for building smart-city by using worldview images. IETE J. Res. 69, 5748–5754. doi: 10.1080/03772063.2022.2163928

[B29] Reddi S. J. Kale S. Kumar S. (2019). On the Convergence of Adam and Beyond.

[B30] Rumelhart D. E. Hinton G. E. Williams R. J. (1986). Learning representations by back-propagating errors. Nature 323, 533–536. doi: 10.1038/323533a0

[B31] Samuel K. Atobatele R. (2019). Land use/cover change and urban sustainability in a medium-sized city. Int. J. Sustain. Soc. 11, 13. doi: 10.1504/IJSSOC.2019.101961

[B32] Sandbhor P. Singh T. Kalshettey M. (2022). Spatiotemporal change in urban landscape and its effect on behavior of diurnal temperature range: a case study of Pune district, India. Environ. Dev. Sustain. 24, 646–665. doi: 10.1007/s10668-021-01461-6

[B33] Selvaraj R. Amali D. G. B. (2024). A new texture aware—seed demand enhanced simple non-iterative clustering (ESNIC) segmentation algorithm for efficient land use and land cover mapping on remote sensing images. IEEE Access 12, 121208–121222. doi: 10.1109/ACCESS.2024.3519612

[B34] Talukdar S. Singha P. Mahato S. Pal S. Liou Y.-A. Rahman A. . (2020). Land-use land-cover classification by machine learning classifiers for satellite observations—a review. Remote Sens. 12:1135. doi: 10.3390/rs12071135

[B35] Tan H. H. Lim K. H. (2019). “Vanishing gradient mitigation with deep learning neural network optimization,” in 2019 7th International Conference on Smart Computing and Communications (ICSCC) (Sarawak: IEEE), 1–4. doi: 10.1109/ICSCC.2019.8843652

[B36] Taye M. M. (2023). Theoretical understanding of convolutional neural network: Concepts, architectures, applications, future directions. Computation 11:52. doi: 10.3390/computation11030052

[B37] Teja J. Vaddi R. Lakshmika K. Nirupama P. (2024). “Land use and land cover change detection using google earth engine,” in 2024 International Conference on Inventive Computation Technologies (ICICT) (Lalitpur: IEEE), 2034–2038. doi: 10.1109/ICICT60155.2024.10544386

[B38] U.S. Geological Survey (2019). Landsat 8 (L8) Data Users Handbook. Department of the Interior. Reston, VA: U.S. Geological Survey.

[B39] Wei X. Zhang W. Zhang Z. Huang H. Meng L. (2023). Urban land use land cover classification based on gf-6 satellite imagery and multi-feature optimization. Geocarto Int. 38:2236579. doi: 10.1080/10106049.2023.2236579

[B40] Whiteside T. Ahmad W. (2005). “A comparison of object-oriented and pixel-based classification methods for mapping land cover in Northern Australia,” in Proceedings of SSC2005 Spatial Intelligence, innovation and Praxis: The National Biennial Conference of the Spatial Sciences Institute, 1225–1231.

[B41] Xie C. Zhu H. Fei Y. (2021). Deep coordinate attention network for single image super-resolution. IET Image Process. 16:12364. doi: 10.1049/ipr2.12364

[B42] Zhao Q. Yu L. Du Z. Peng D. Hao P. Zhang Y. . (2022). An overview of the applications of earth observation satellite data: impacts and future trends. Remote Sens. 14:1863. doi: 10.3390/rs14081863

[B43] Zhao S. Tu K. Ye S. Tang H. Hu Y. Xie C. . (2023). Land use and land cover classification meets deep learning: a review. Sensors 23:8966. doi: 10.3390/s23218966 37960665 PMC10649958

